# Supplementary material for: Unraveling the impact of AXIN1 mutations on HCC development: Insights from CRISPR/Cas9 repaired AXIN1-mutant liver cancer cell lines
Source: PLoS One. 2024 Jun 7;19(6):e0304607. doi: 10.1371/journal.pone.0304607 (PMC11161089; doi:10.1371/journal.pone.0304607)
Supplement: S1 Fig — A) Baseline information of HCC cell lines used in this study, including differentiation subtype as reported by Caruso et al. [28], puromycin concentration used to select clones, and the type of AXIN1 mutation. Microscopic images are obtained from the Zucman lab website (https://lccl.zucmanlab.com/hcc/cellLines). (B) Original sequence chromatograms depicting the mutations observed in each cell line. (PDF) [file pone.0304607.s001.pdf]

A

Cell line	AXIN1 mutation		Differentiation	Mycoplasma	Puromycin	Microscopic image
JHH6	**GC-TT replacement in exon 2 leads to a missense mutation (E5D) and a stop codon (Q6*)**	**p.E5D+Q6***	**Mesenchymal-like**	**negative**	**2 µg/ml**	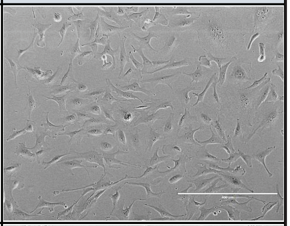
JHH7	**Homozygous deletion in exon 2 of the following : TGACCAAGATGGGATAAGCCTGTTCAGGACTT TCCTGAAGCAGGA**	**p.D94_Q108del**	**Hepatoblast-like**	**negative**	**1 µg/ml**	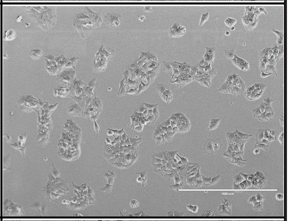
Hep 3B 2.1-7	**Homozygous CGA-TGA replacement (c.436C>T) in exon 2 leads to direct stop codon**	**p.R146***	**Hepatoblast-like**	**negative**	**1 µg/ml**	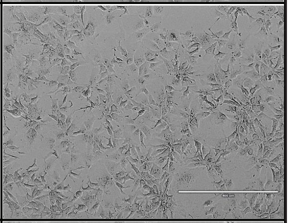
HuH1	**Homozygous GGGAGCCAGTCAAC deletion in exon 3 Sequence is replaced by one T**	**p.R298Lfs*112**	**Hepatoblast-like**	**negative**	**2-4 µg/ml**	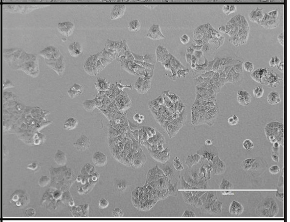
SNU423	**Homozygous CCATACAGGATCC deletion in exon4**	**p.P345Vfs*65**	**Mesenchymal-like**	**negative**	**2 µg/ml**	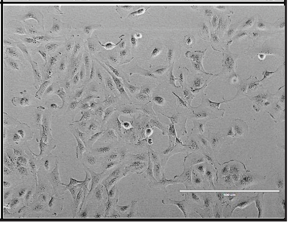

B

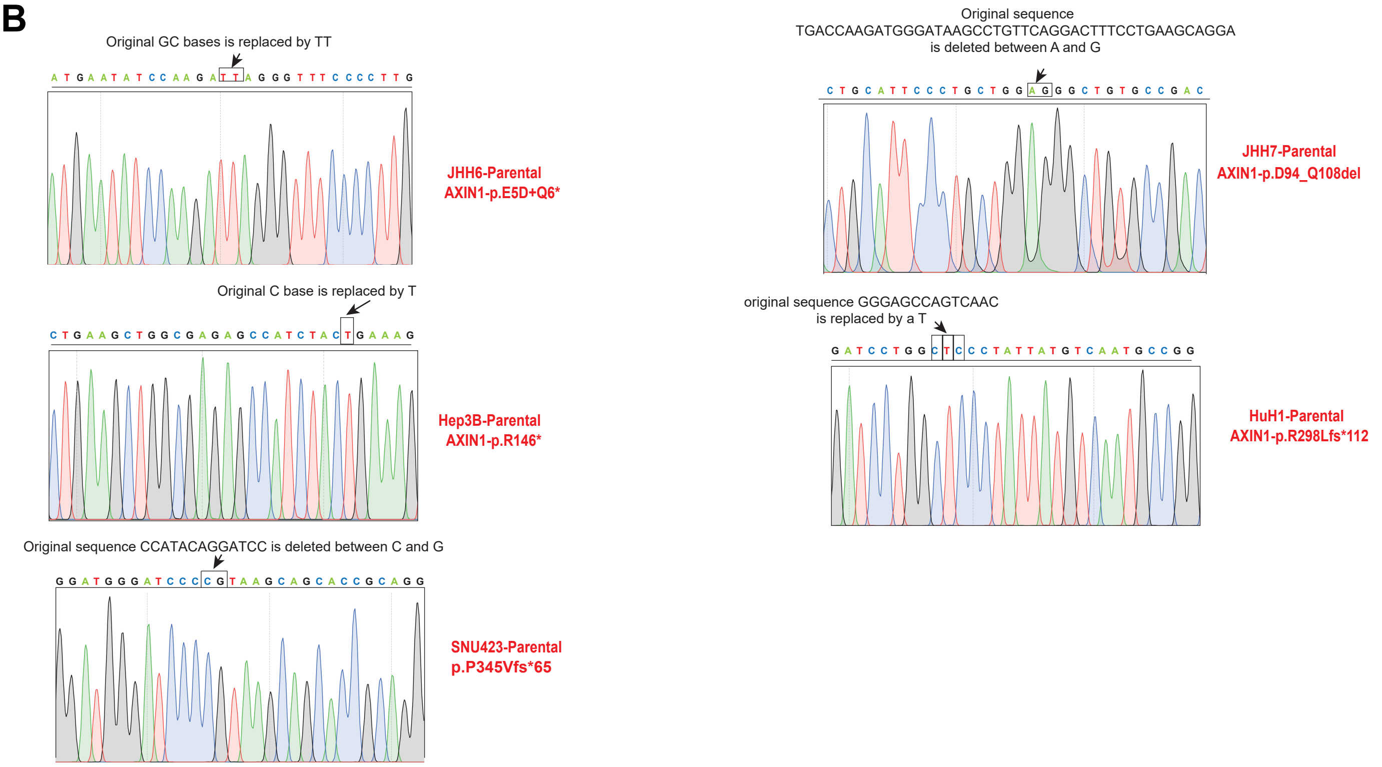

Supplementary Fig S1. (A) Baseline information of HCC cell lines used in this study, including differentiation subtype as reported by Caruso et al.[28], puromycin concentration used to select clones, and the type of AXIN1 mutation. Microscopic images are obtained from the Zucman lab website (https://lccl.zucmanlab.com/hcc/cellLines). (B) Original sequence chromatograms depicting the mutations observed in each cell line.
