# Supplementary material for: Unraveling the impact of AXIN1 mutations on HCC development: Insights from CRISPR/Cas9 repaired AXIN1-mutant liver cancer cell lines
Source: PLoS One. 2024 Jun 7;19(6):e0304607. doi: 10.1371/journal.pone.0304607 (PMC11161089; doi:10.1371/journal.pone.0304607)
Supplement: S2 Fig — (A) When expressed in HEK293T cells it leads to a strong increase in β-catenin reporter activity comparable to adding exogenous Wnt3a ligand, while wild-type AXIN1 has no effect. This shows that the D94_Q108del variant protein interferes with β-catenin regulation in a dominant manner when overexpressed. (B) A co-immunoprecipitation experiment with GFP-tagged APC (aa 1199–2167) shows that the D94_Q108del variant is unable to bind APC. Statistical significance was analyzed using a Mann-Whitney test (****P < 0.0001). These data have also been described elsewhere (ref 18), but are reproduced here for clarity. (PDF) [file pone.0304607.s002.pdf]

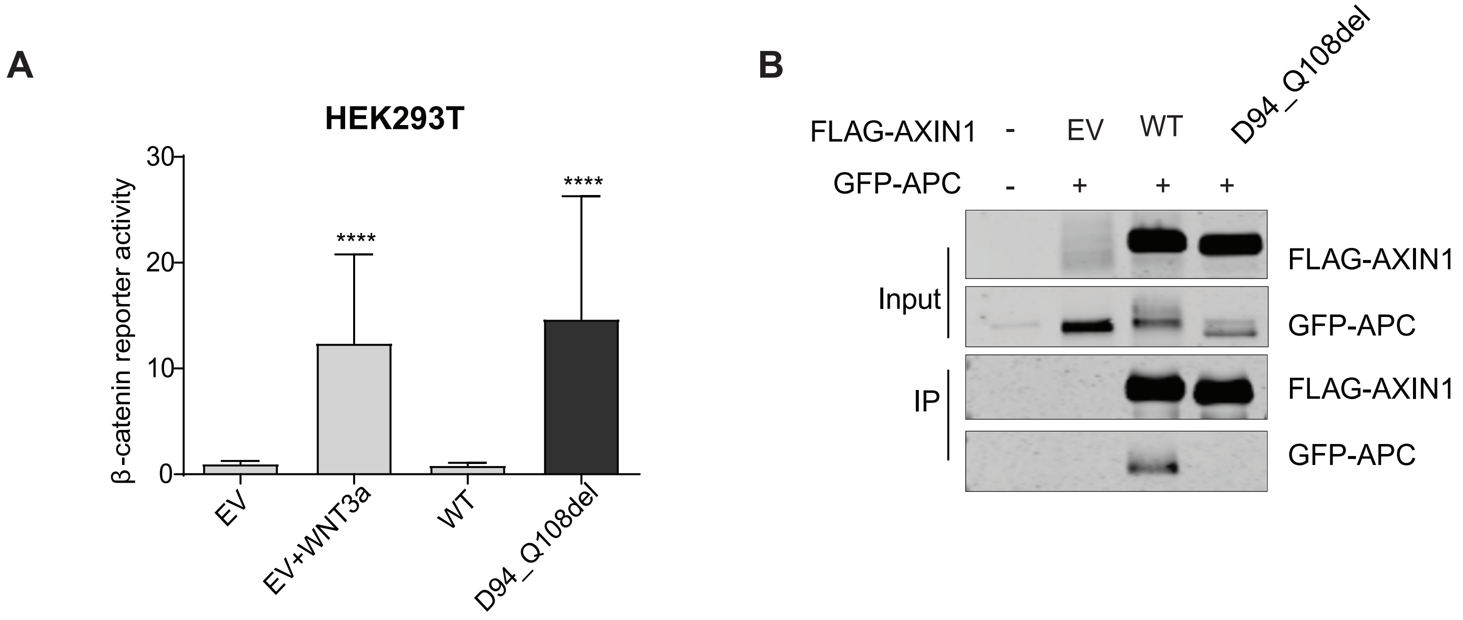

Supplementary Fig S2. An AXIN1 expression construct was generated that carries the same D94_Q108 deletion as present in the JHH7 cell line. (A) When expressed in HEK293T cells it leads to a strong increase in β-catenin reporter activity comparable to adding exogenous Wnt3a ligand, while wild-type AXIN1 has no effect. This shows that the D94_Q108del variant protein interferes with β-catenin regulation in a dominant manner when overexpressed. (B) A co-immunoprecipitation experiment with GFP-tagged APC (aa 1199-2167) shows that the D94_Q108del variant is unable to bind APC. Statistical significance was analyzed using a Mann-Whitney test (****P < 0.0001). These data have also been described elsewhere (ref 18), but are reproduced here for clarity.
