# Supplementary material for: Unraveling the impact of AXIN1 mutations on HCC development: Insights from CRISPR/Cas9 repaired AXIN1-mutant liver cancer cell lines
Source: PLoS One. 2024 Jun 7;19(6):e0304607. doi: 10.1371/journal.pone.0304607 (PMC11161089; doi:10.1371/journal.pone.0304607)
Supplement: S4 Fig — qPCR was used to measure NOTUM expression levels. With the exception of JHH6-repaired-E12, the expression level of NOTUM was found to be lower in the AXIN1-repaired clones compared to the parental cells. The data were normalized to the housekeeping gene GAPDH, with the parental values set to 1. The statistical significance of the results was analyzed using the Mann-Whitney test, with the level of significance indicated as follows: (*P < 0.05, **P < 0.01, ***P < 0.001, ***P < 0.0001). (PDF) [file pone.0304607.s004.pdf]

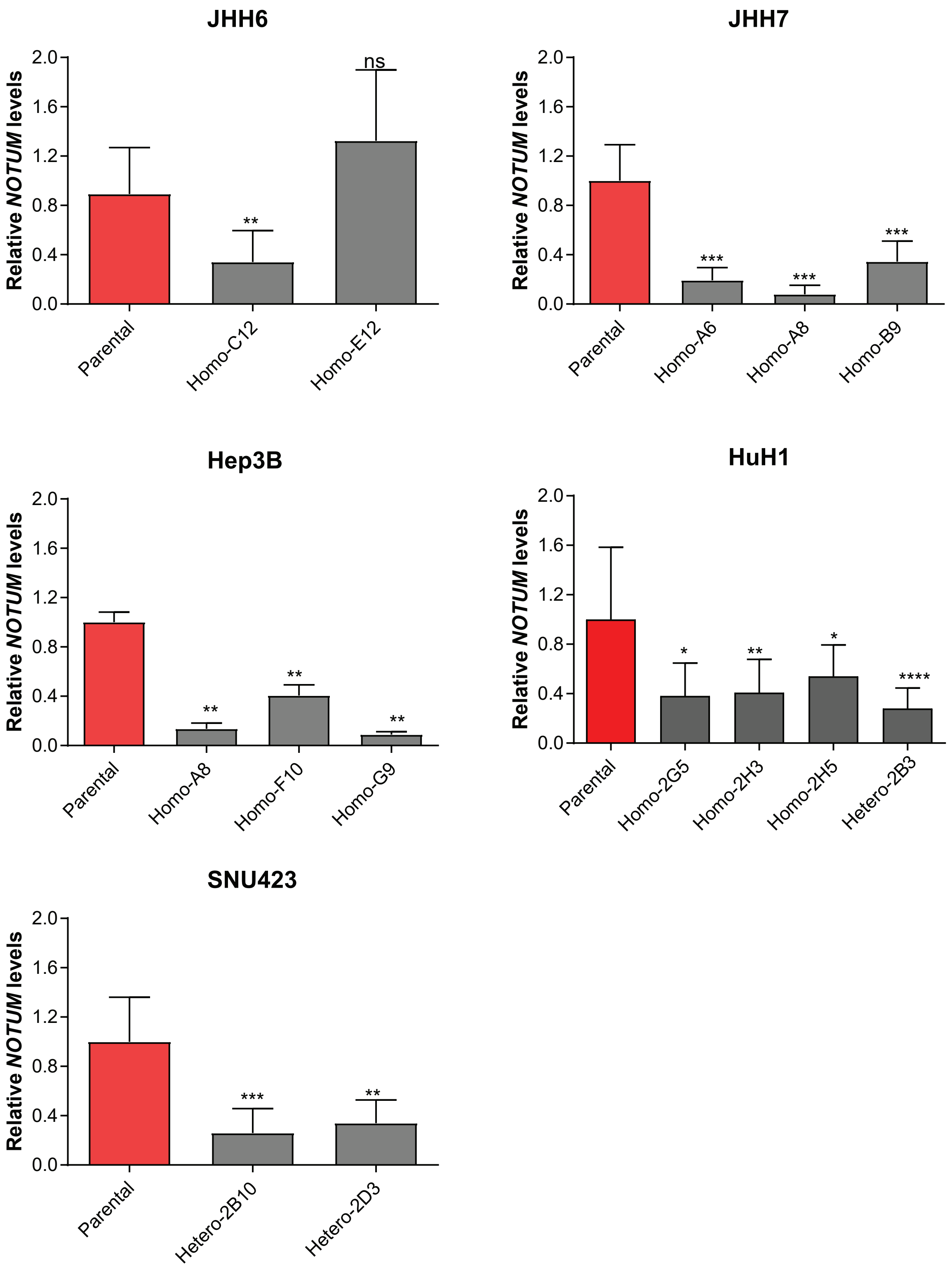

Supplementary Fig S4. Expression levels of *NOTUM* mRNA in AXIN1-repaired clones. qPCR was used to measure *NOTUM* expression levels. With the exception of JHH6-repaired-E12, the expression level of NOTUM was found to be lower in the AXIN1-repaired clones compared to the parental cells. The data were normalized to the housekeeping gene *GAPDH*, with the parental values set to 1. The statistical significance of the results was analyzed using the Mann-Whitney test, with the level of significance indicated as follows: (*$P < 0.05$, **$P < 0.01$, ***$P < 0.001$, ***$P < 0.0001$).
