# Supplementary material for: Unraveling the impact of AXIN1 mutations on HCC development: Insights from CRISPR/Cas9 repaired AXIN1-mutant liver cancer cell lines
Source: PLoS One. 2024 Jun 7;19(6):e0304607. doi: 10.1371/journal.pone.0304607 (PMC11161089; doi:10.1371/journal.pone.0304607)
Supplement: S5 Fig — To this aim, a β-catenin reporter assay was performed. The β-catenin reporter activities are presented as WRE/CMV-Renilla ratios (mean ± SD, n = 3, two independent experiments). The values depicted here, were used to determine the siAXIN2/siControl ratios shown in Fig 2D. All values were scaled to log10. (PDF) [file pone.0304607.s005.pdf]

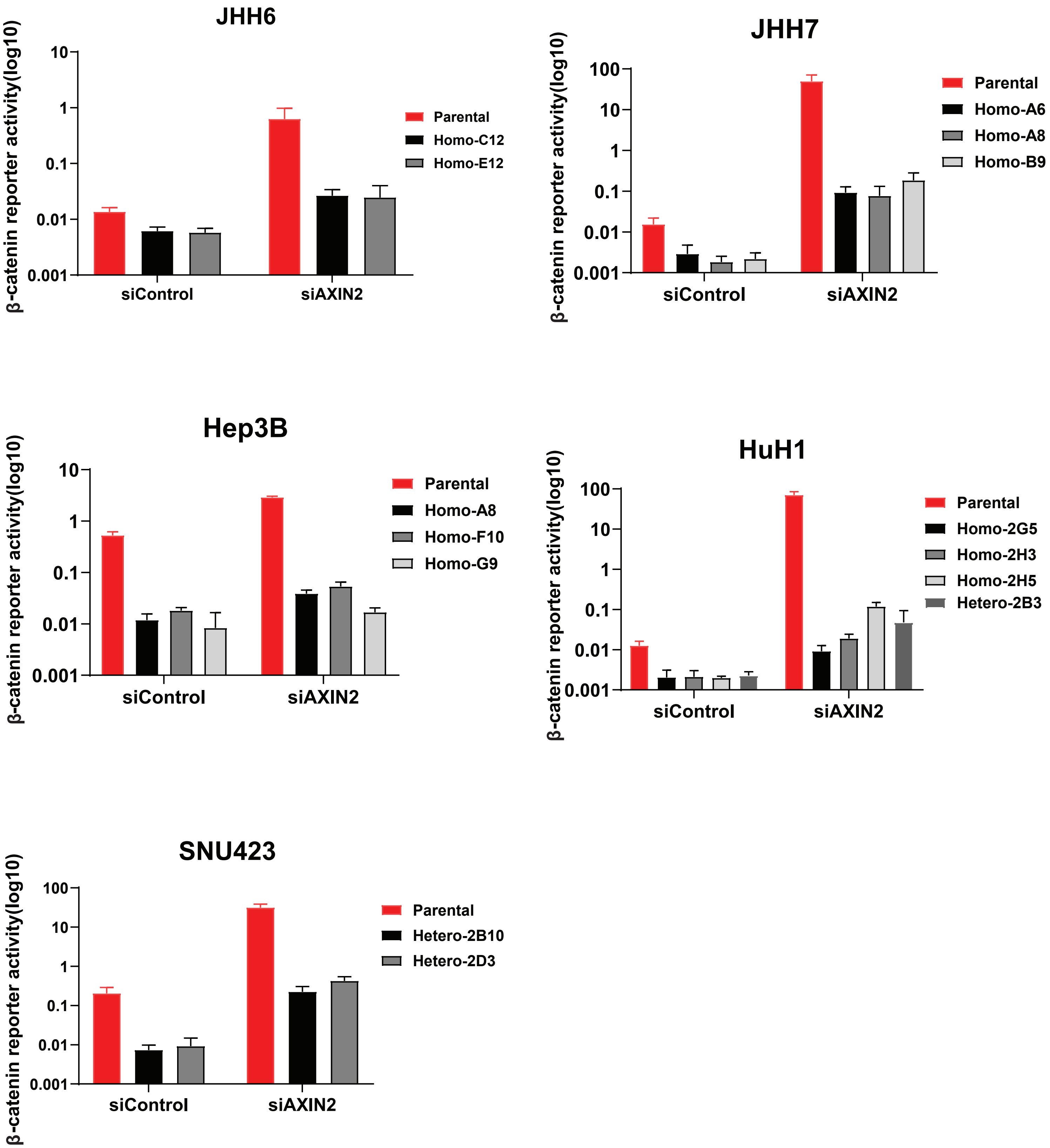

Supplementary Fig S5. The impact of siAXIN2 on the β-catenin signaling was evaluated in both parental and repaired clones of the HCC cell line. To this aim, a β-catenin reporter assay was performed. The β-catenin reporter activities are presented as WRE/CMV-Renilla ratios (mean ± SD, n=3, two independent experiments). The values depicted here, were used to determine the siAXIN2/siControl ratios shown in Figure 2D. All values were scaled to log10.
