# Supplementary material for: Unraveling the impact of AXIN1 mutations on HCC development: Insights from CRISPR/Cas9 repaired AXIN1-mutant liver cancer cell lines
Source: PLoS One. 2024 Jun 7;19(6):e0304607. doi: 10.1371/journal.pone.0304607 (PMC11161089; doi:10.1371/journal.pone.0304607)
Supplement: S7 Fig — (A) The RNA sequencing data were subjected to principal component analysis, which clearly separated the samples into five distinct groups based on cell line identity. (B) Volcano plots for each cell line separately, showing genes significantly altered at least log2 fold change of 1 in expression (p <0.01). (PDF) [file pone.0304607.s007.pdf]

A

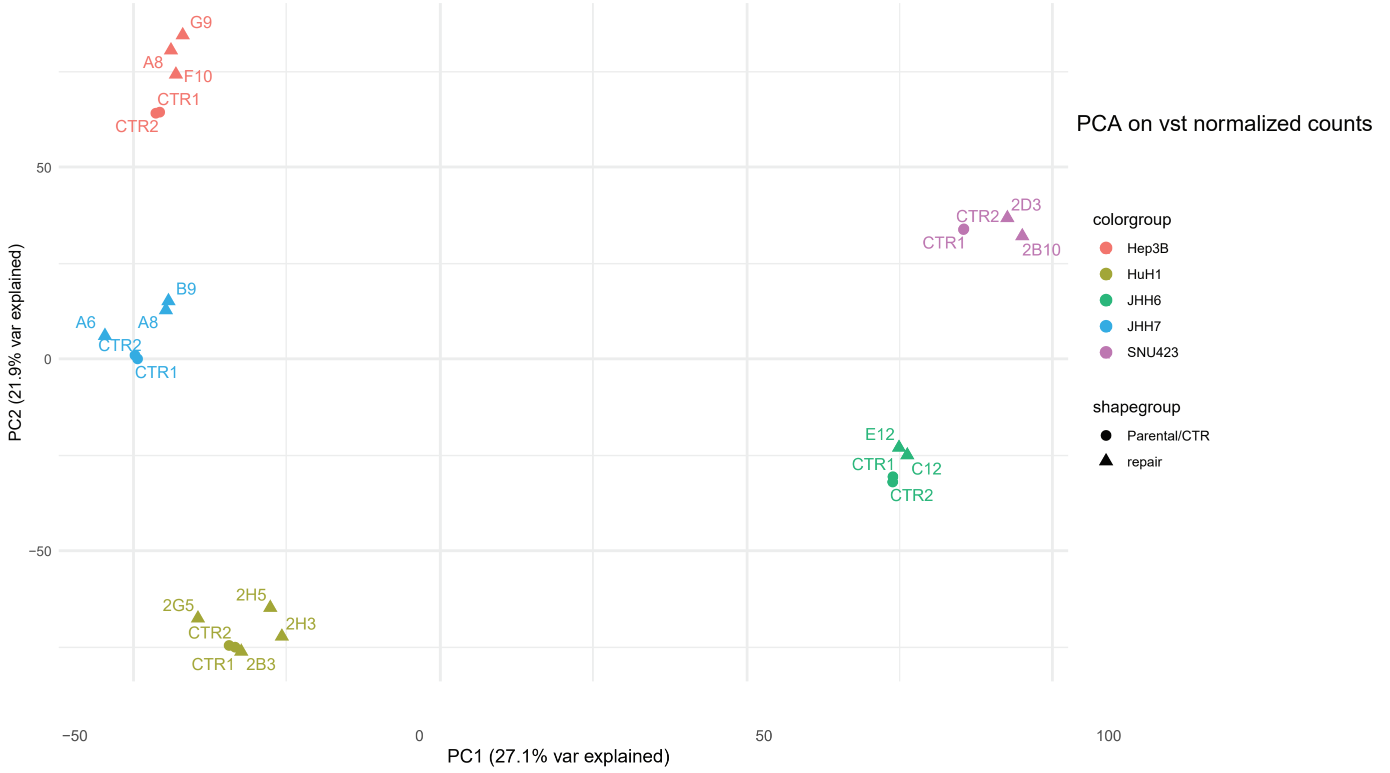

B

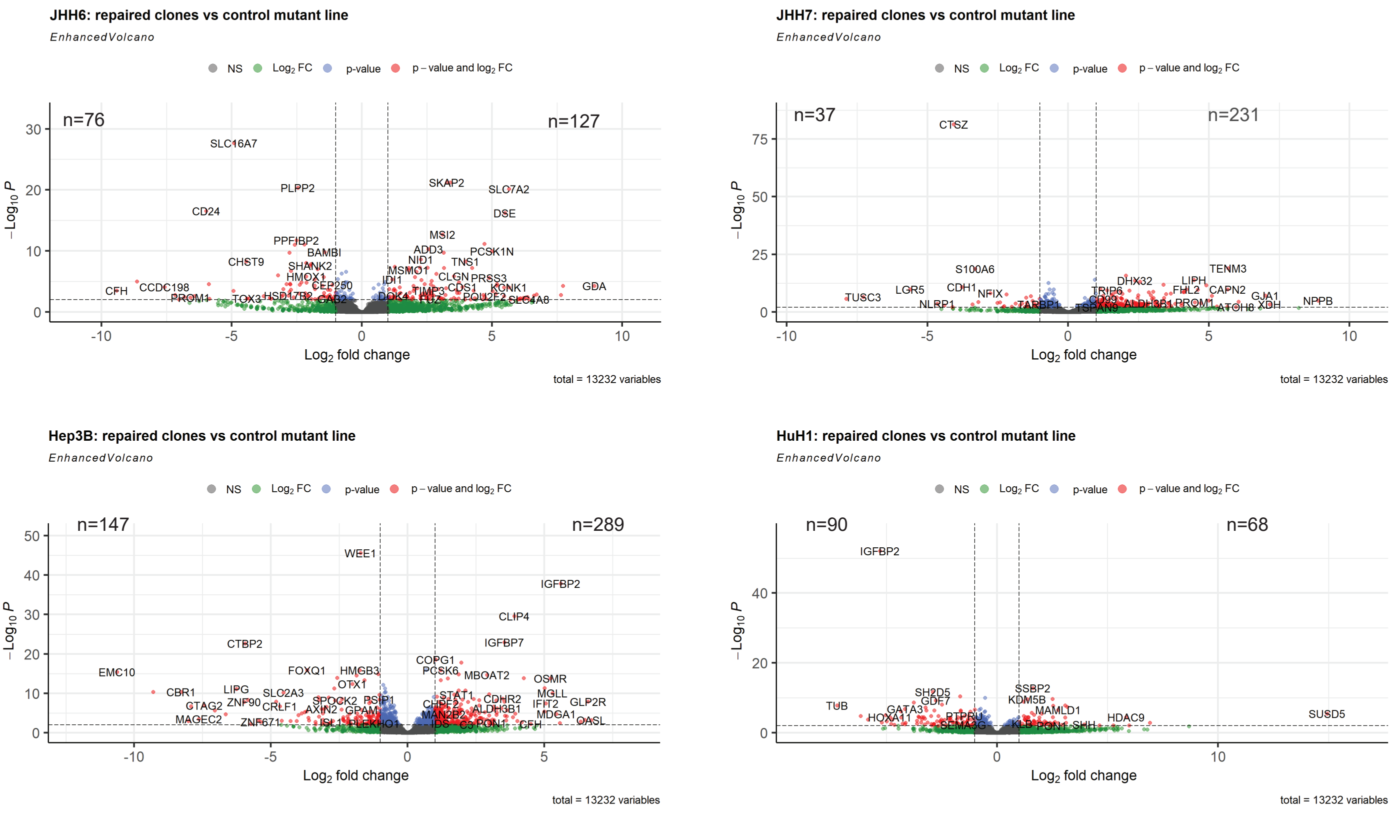

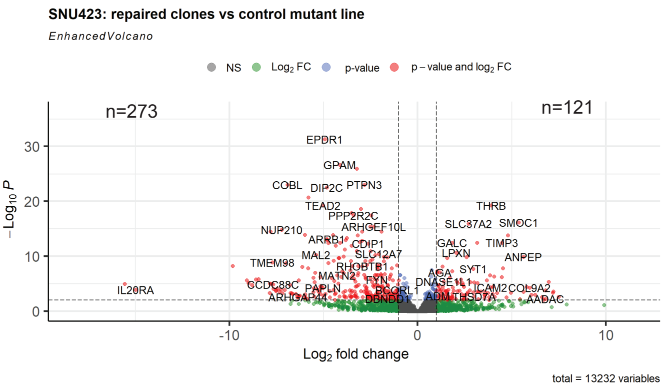

Supplementary Fig S7. RNA-seq analysis for AXIN1-repaired clones and their corresponding parental HCC cell lines. (A) The RNA sequencing data were subjected to principal component analysis, which clearly separated the samples into five distinct groups based on cell line identity. (B) Volcano plots for each cell line separately, showing genes significantly altered at least log2 fold change of 1 in expression (p < 0.01).
