# Supplementary material for: Unraveling the impact of AXIN1 mutations on HCC development: Insights from CRISPR/Cas9 repaired AXIN1-mutant liver cancer cell lines
Source: PLoS One. 2024 Jun 7;19(6):e0304607. doi: 10.1371/journal.pone.0304607 (PMC11161089; doi:10.1371/journal.pone.0304607)
Supplement: S8 Fig — (PDF) [file pone.0304607.s008.pdf]

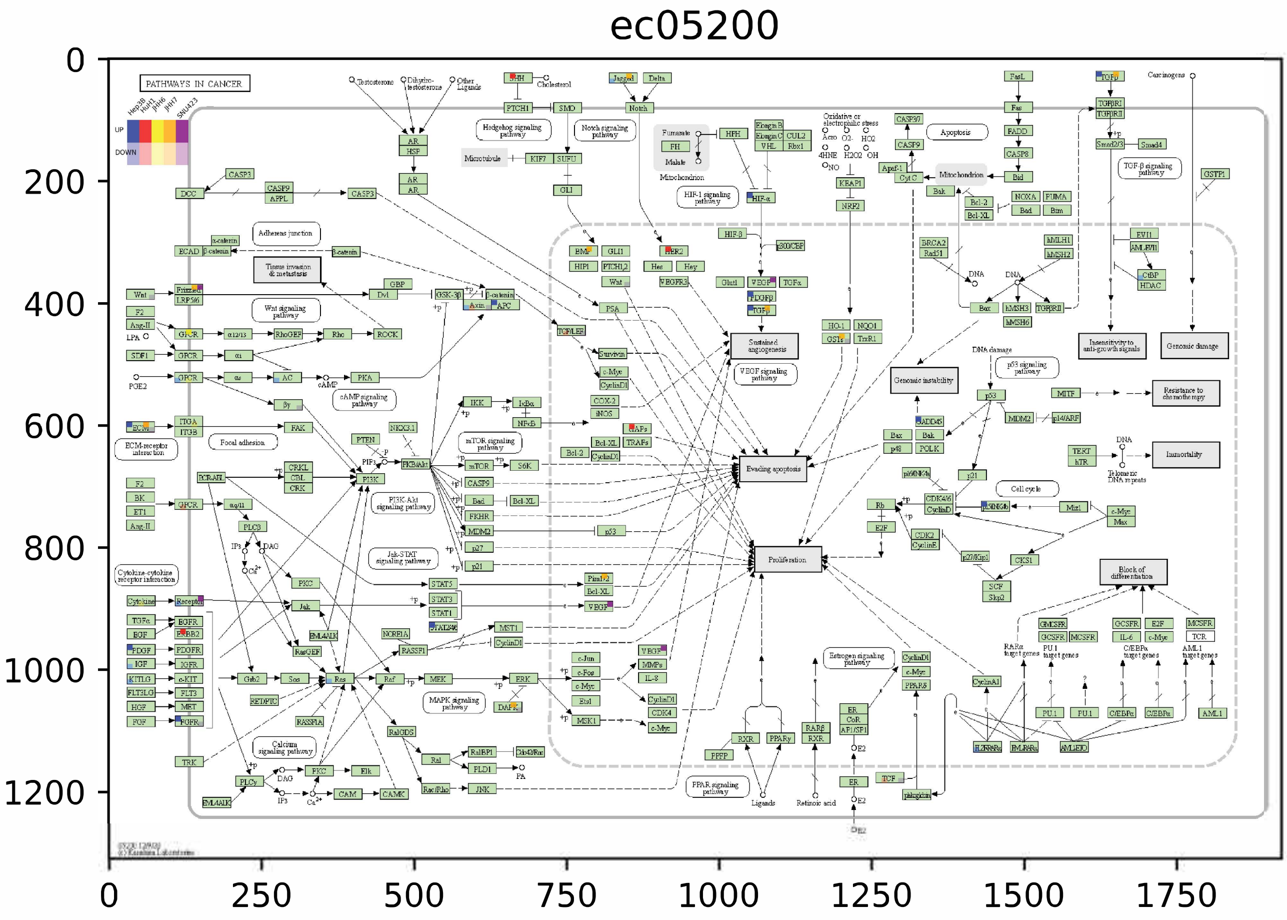

Supplementary Fig S8.

A KEGG analysis does not reveal a pathway consistently altered in all cell lines
