# Supplementary material for: Unraveling the impact of AXIN1 mutations on HCC development: Insights from CRISPR/Cas9 repaired AXIN1-mutant liver cancer cell lines
Source: PLoS One. 2024 Jun 7;19(6):e0304607. doi: 10.1371/journal.pone.0304607 (PMC11161089; doi:10.1371/journal.pone.0304607)
Supplement: S9 Fig — The data was normalized to the housekeeping gene GAPDH (mean ± SD, n = 2, two independent experiments). Additionally, the data was further normalized to the corresponding parental cell line, with the parental expression set to 1. Statistical significance for all experiments was analyzed using a Mann-Whitney test (*P < 0.05). (PDF) [file pone.0304607.s009.pdf]

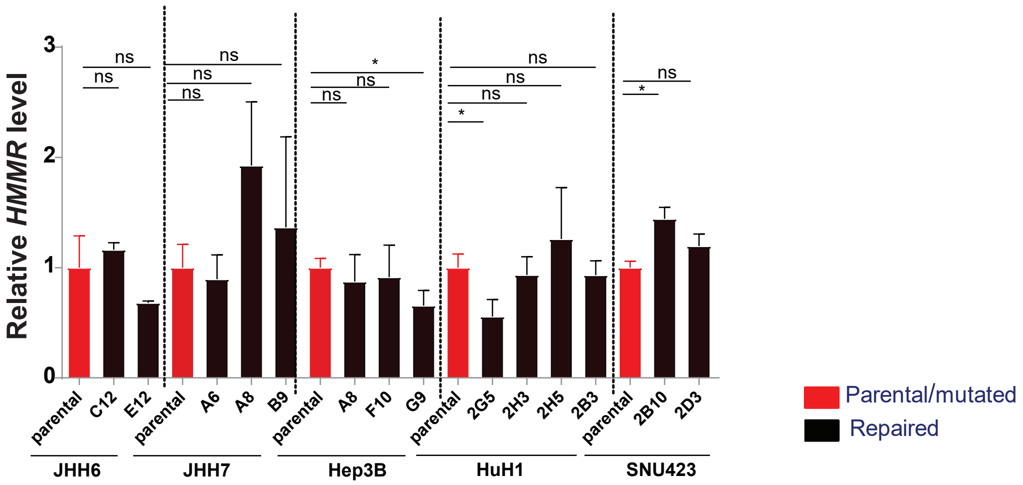

Supplementary Fig S9. QRT-PCR assay shows the relative *HMMR* mRNA expression levels. The data was normalized to the housekeeping gene *GAPDH* (mean ± SD, n=2, two independent experiments). Additionally, the data was further normalized to the corresponding parental cell line, with the parental expression set to 1. Statistical significance for all experiments was analyzed using a Mann-Whitney test (*$P < 0.05$).
