# Supplementary material for: Unraveling the impact of AXIN1 mutations on HCC development: Insights from CRISPR/Cas9 repaired AXIN1-mutant liver cancer cell lines
Source: PLoS One. 2024 Jun 7;19(6):e0304607. doi: 10.1371/journal.pone.0304607 (PMC11161089; doi:10.1371/journal.pone.0304607)
Supplement: S10 Fig — (PDF) [file pone.0304607.s010.pdf]

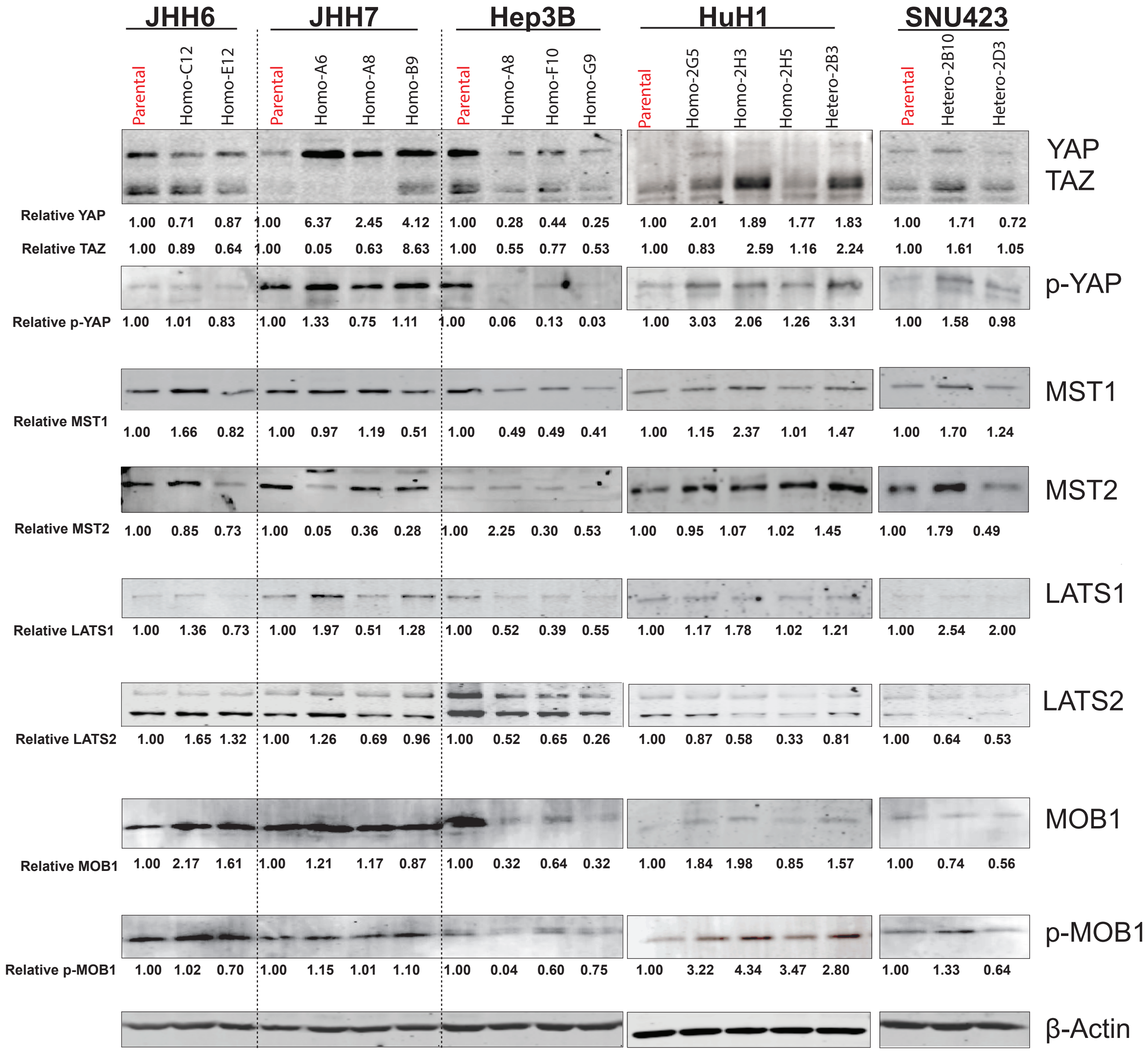

Supplementary Fig S10. Quantification of band intensities for indicated proteins for the immunoblots shown in Figure 6C.
