# Supplementary material for: Unraveling the impact of AXIN1 mutations on HCC development: Insights from CRISPR/Cas9 repaired AXIN1-mutant liver cancer cell lines
Source: PLoS One. 2024 Jun 7;19(6):e0304607. doi: 10.1371/journal.pone.0304607 (PMC11161089; doi:10.1371/journal.pone.0304607)

Supplemental Fig.11. Original immunoblots

Fig.1C

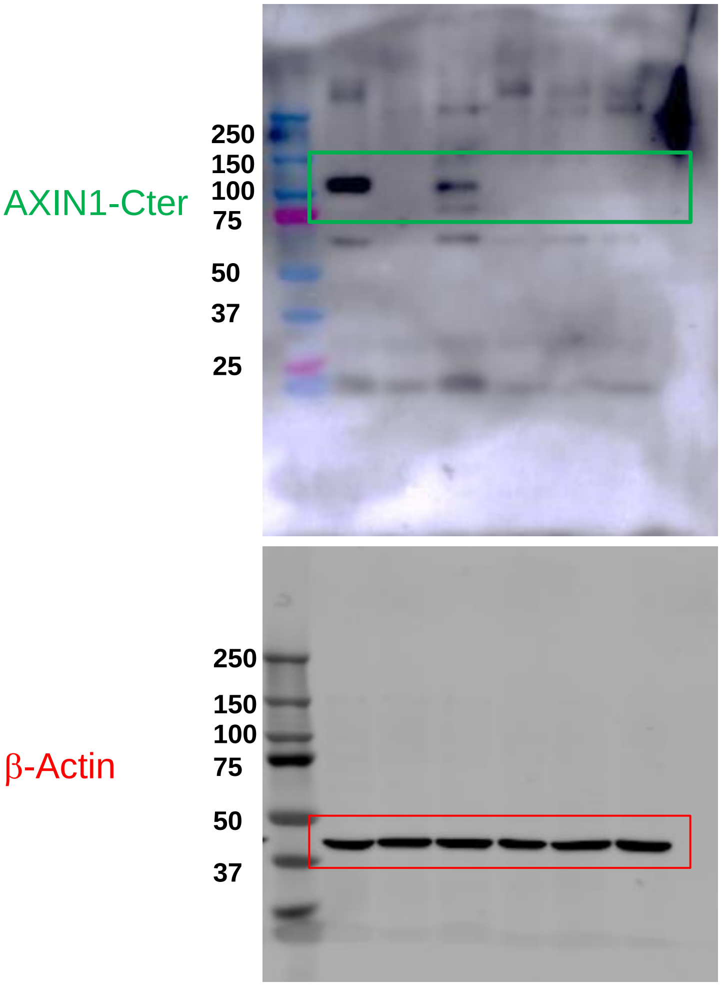

Lane	
1	**Marker**
2	HEK293T
3	JHH6
4	JHH7
5	Hep3B
6	HuH1
7	SNU423

Fig.2A Page1; JHH6 and JHH7

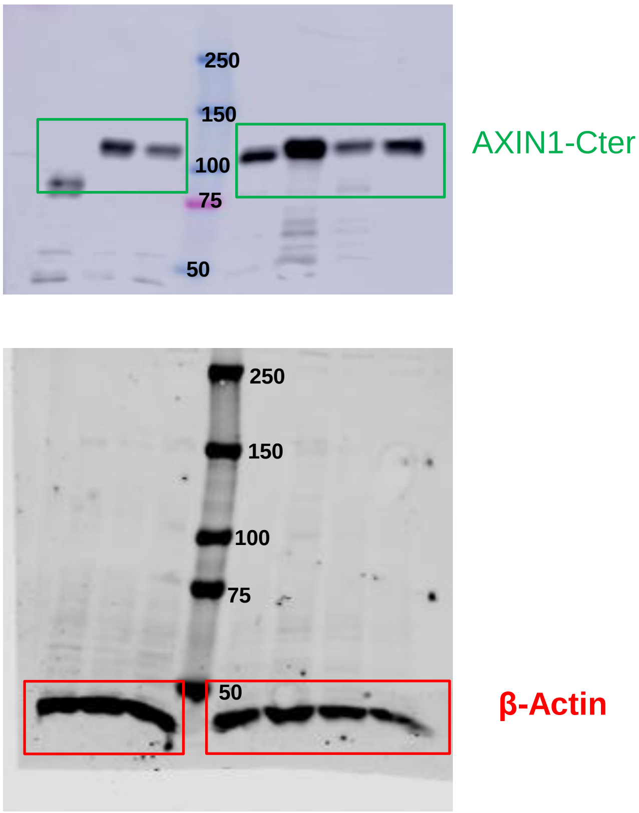

Lane
1. JHH6 parental
2. JHH6 C12
3. JHH6 E12
4. Marker
5. JHH7 parental
6. JHH7 A6
7. JHH7 A8
8. JHH7 B9

Fig.2A Page 2; Hep3B

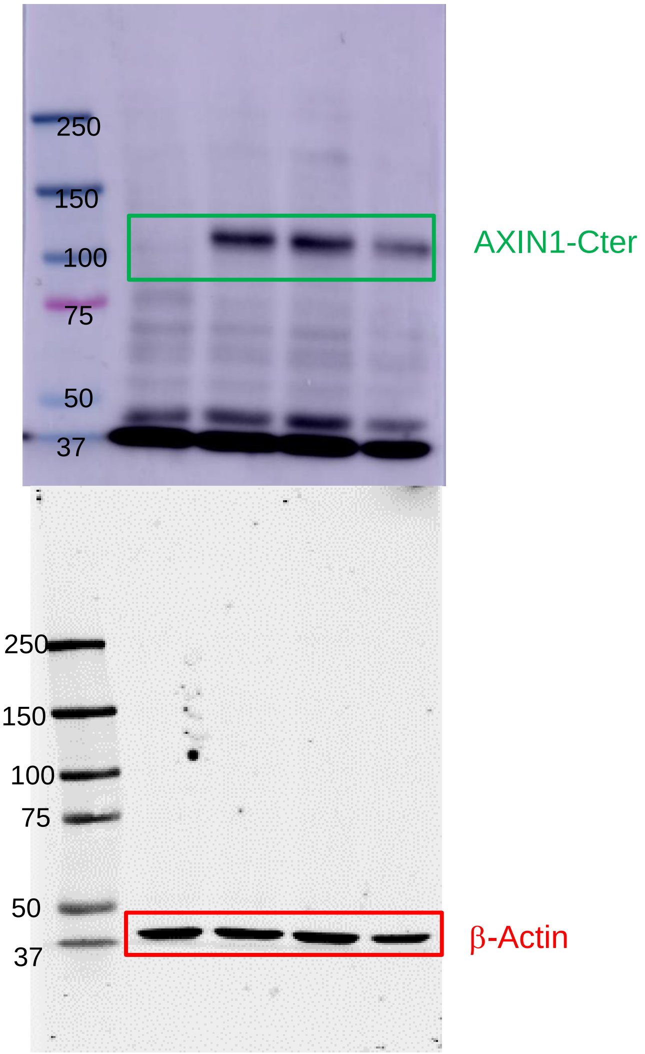

Lane

1. Marker
2. Hep3B-a2 parental
3. Hep3B-Homo-A8
4. Hep3B-Homo-F10
5. Hep3B-Homo-G9

Fig.2A Page 3; HuH1

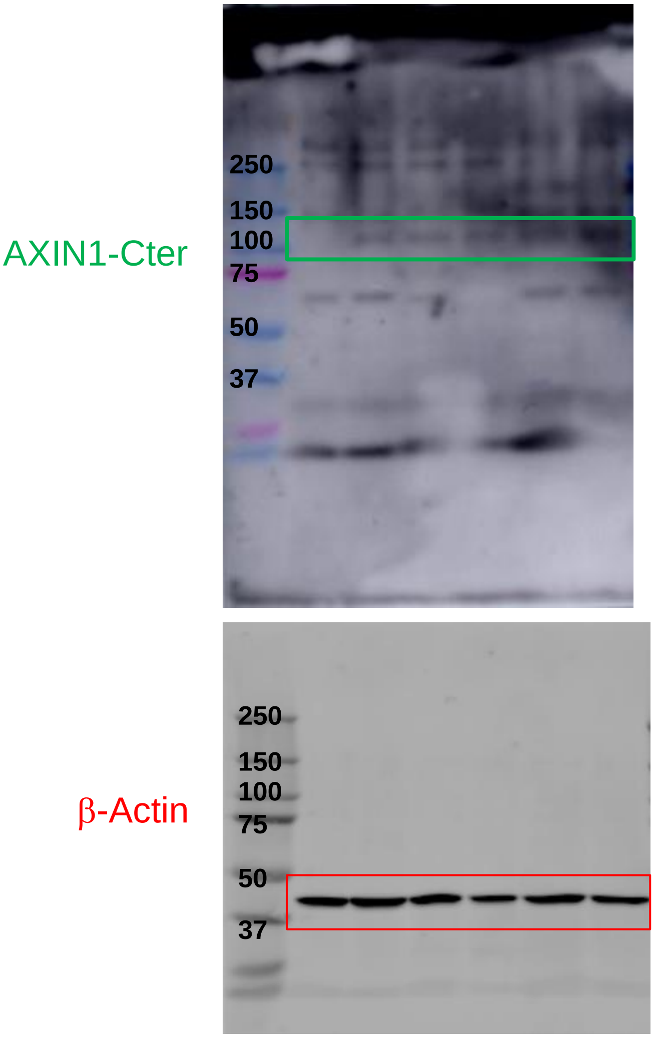

Lane	
1	**Marker**
2	HuH1-Parental
3	HuH1-Homo-2D1
4	HuH1-Homo-2G5
5	HuH1-Homo-2H3
6	HuH1-Homo-2H5
7	HuH1-Homo-2B3

Fig.2A Page 4; SNU423

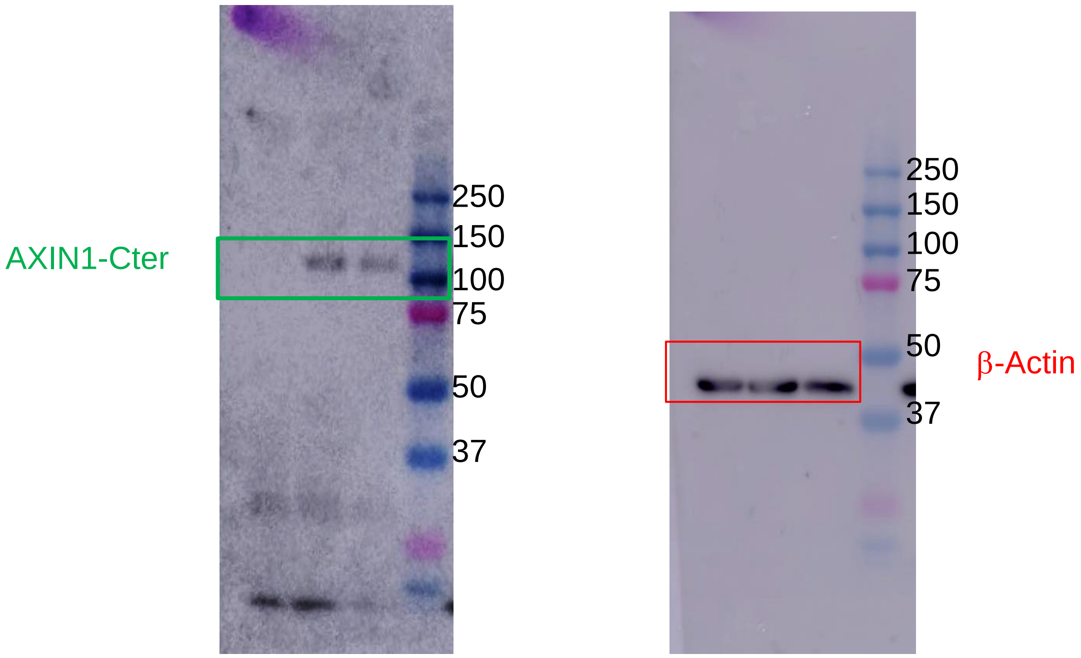

Lane

1	SNU423-Parental
2	SNU423-Hetero-2B10
3	SNU423-Hetero-2D3

Fig.6C page 1

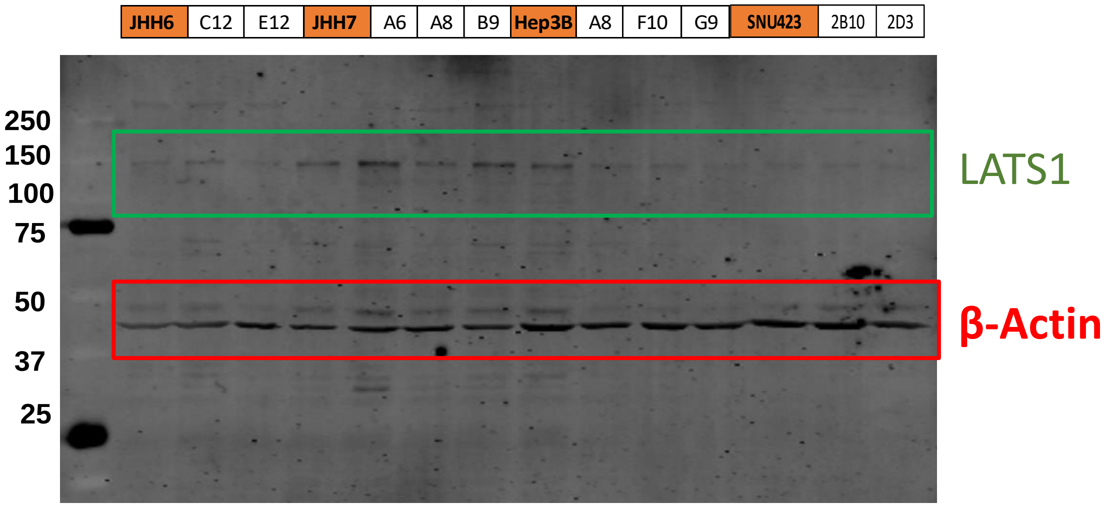
JHH6
C12
E12
JHH7
A6
A8
B9
Hep3B
A8
F10
G9
SNU423
2B10
2D3
250
150
100
75
50
37
25
LATS1
β-Actin

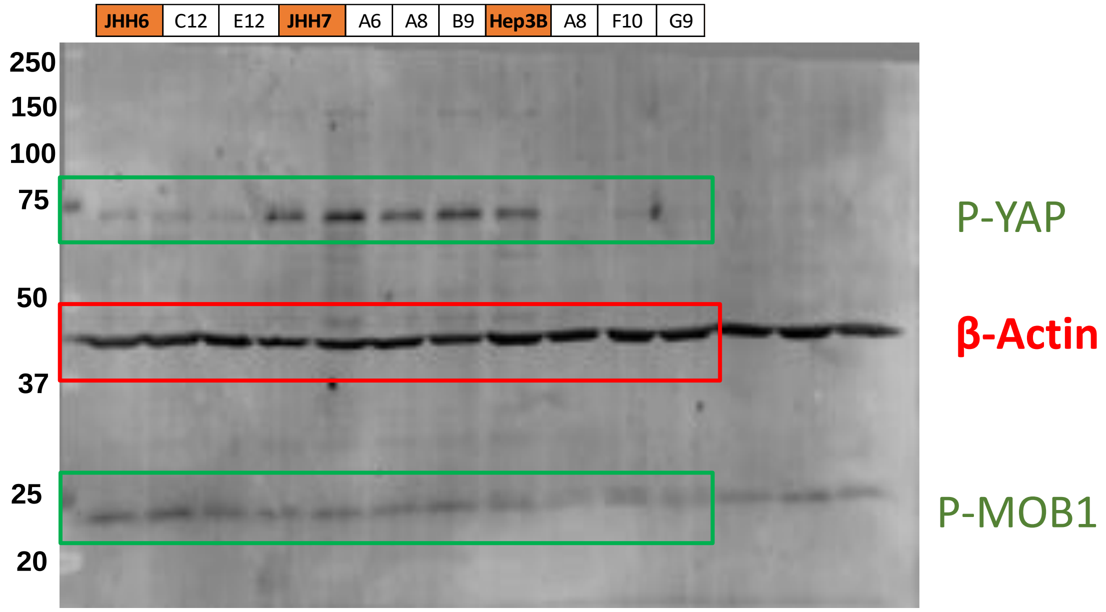
JHH6
C12
E12
JHH7
A6
A8
B9
Hep3B
A8
F10
G9
250
150
100
75
50
37
25
20
P-YAP
β-Actin
P-MOB1

Fig.6C page2

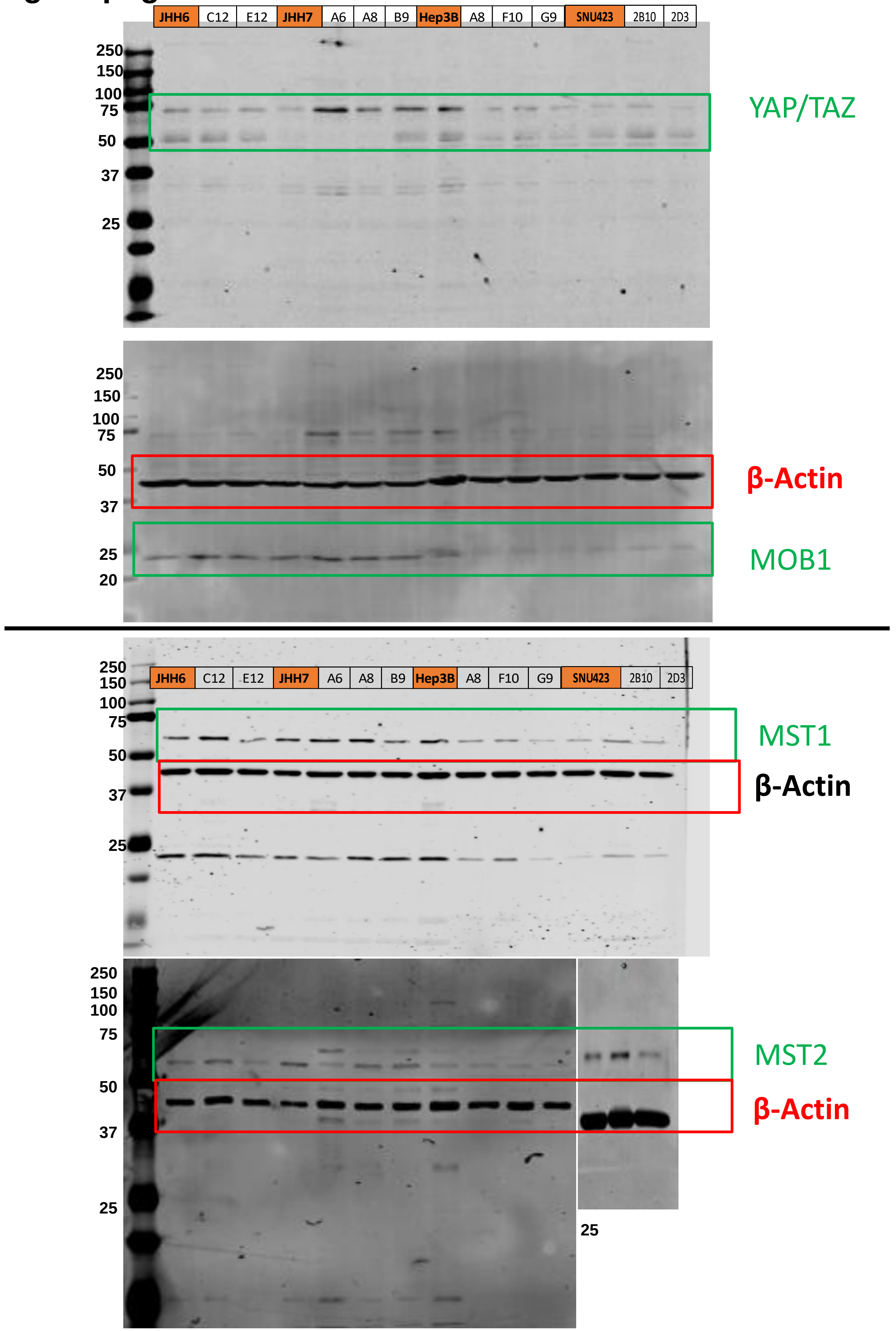
JHH6
C12
E12
JHH7
A6
A8
B9
Hep3B
A8
F10
G9
SNU423
2B10
2D3
250
150
100
75
50
37
25
20
YAP/TAZ
β-Actin
MOB1
MST1
MST2

Fig.6C page 3

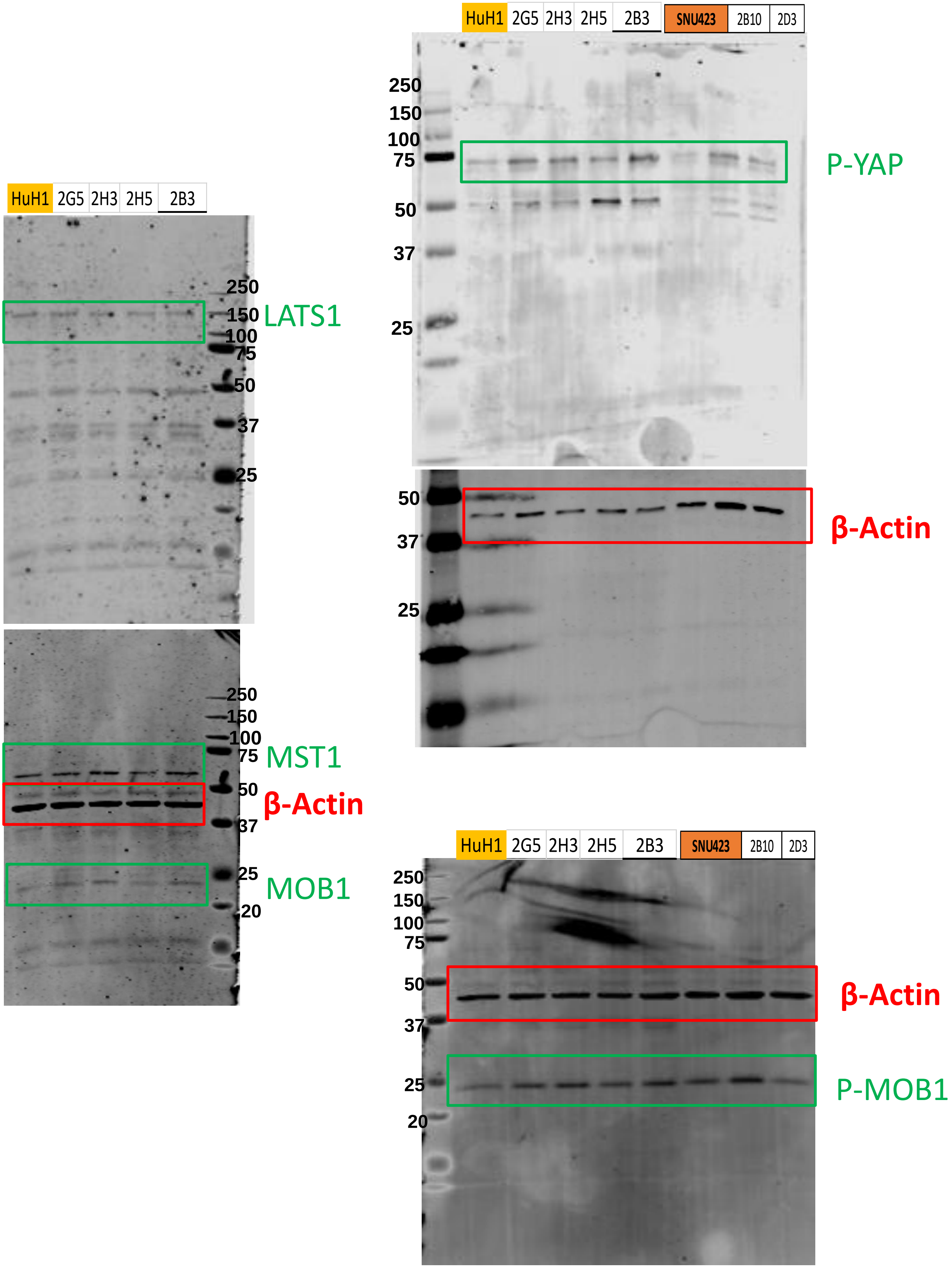
HuH1
2G5
2H3
2H5
2B3
SNU423
2B10
2D3
250
150
100
75
50
37
25
20
LATS1
MST1
β-Actin
MOB1
P-YAP
β-Actin
β-Actin
P-MOB1

Fig.6C page 4

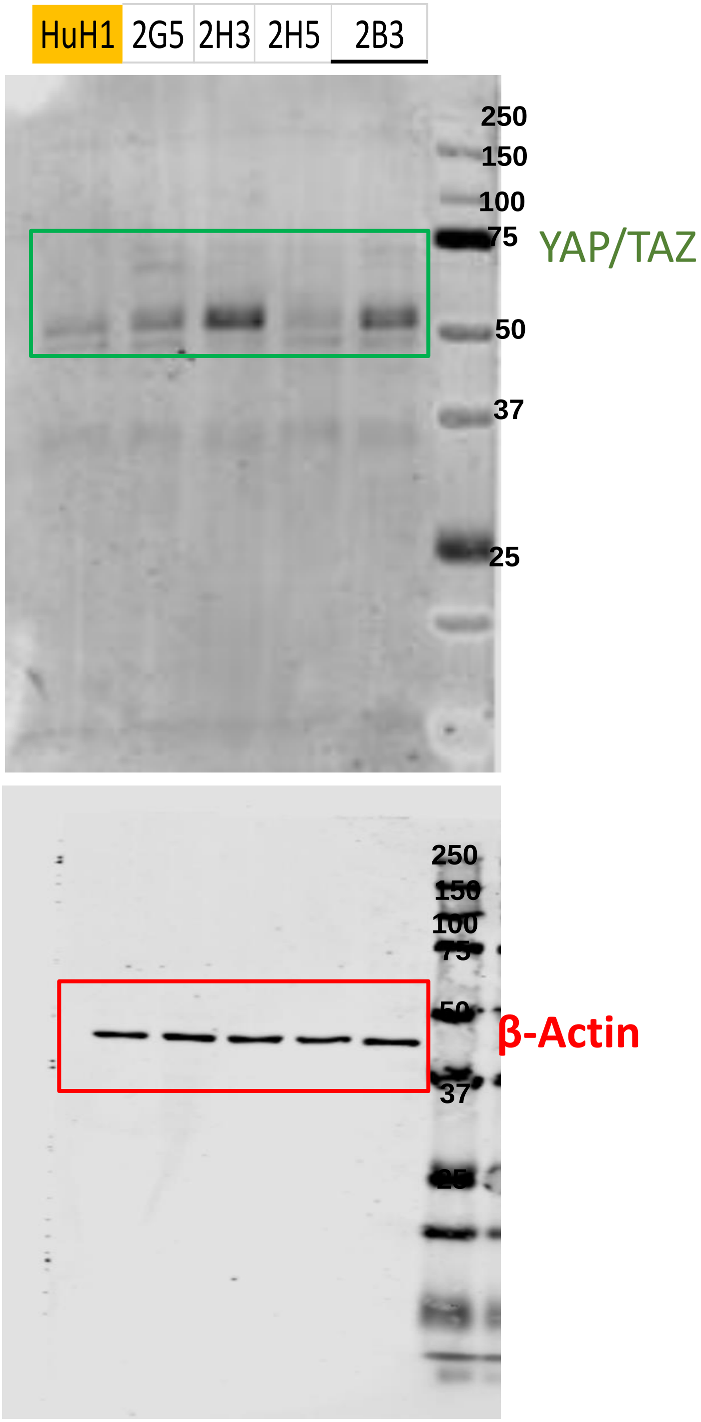
HuH1
2G5
2H3
2H5
2B3
250
150
100
75
50
37
25
YAP/TAZ
250
150
100
75
50
37
25
β-Actin

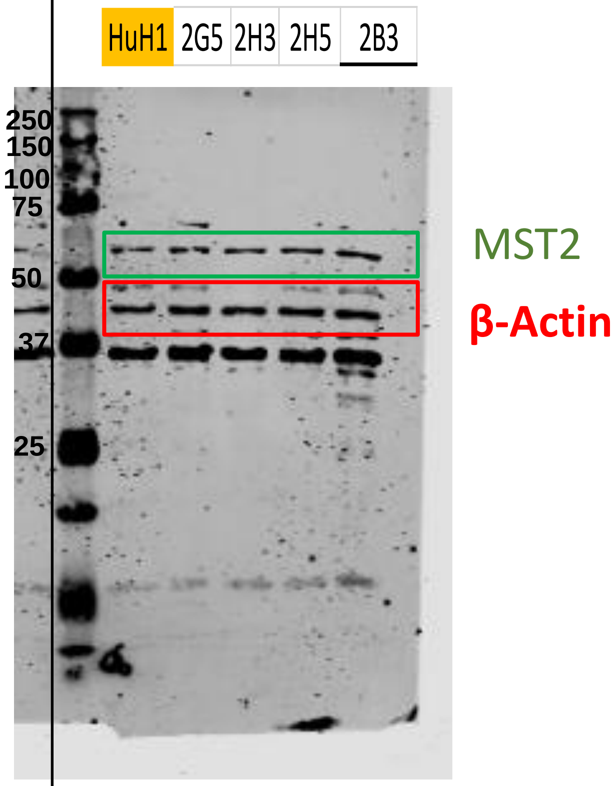
HuH1
2G5
2H3
2H5
2B3
250
150
100
75
50
37
25
MST2
β-Actin

Fig.6C page 5

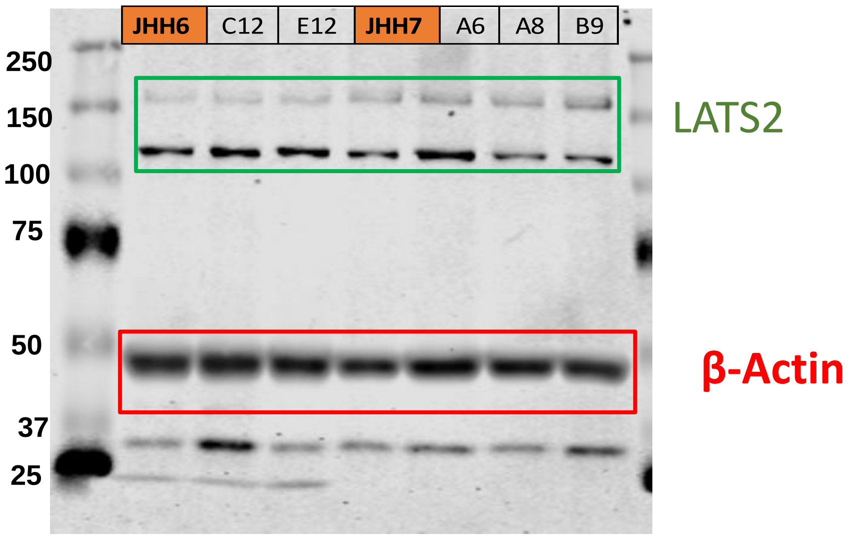

JHH6
C12
E12
JHH7
A6
A8
B9
250
150
100
75
50
37
25
LATS2
β-Actin

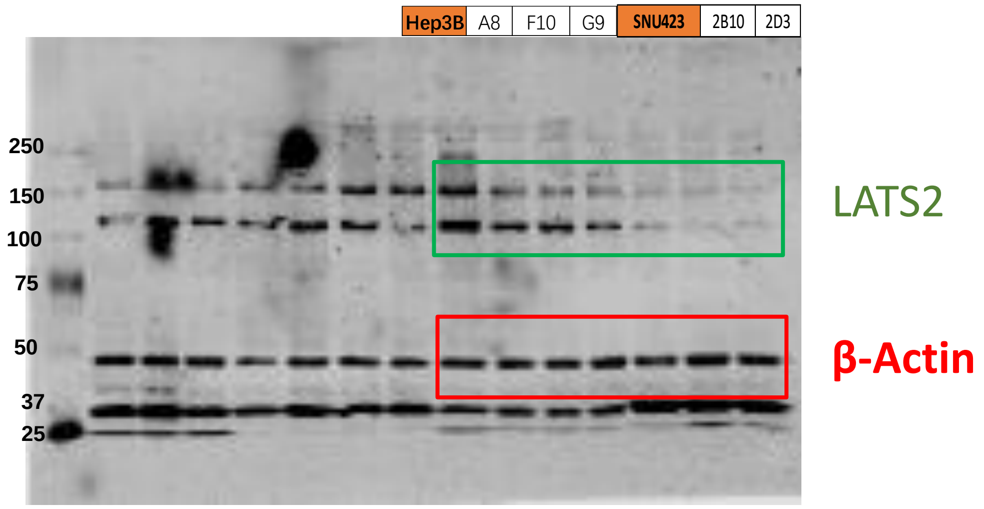

Hep3B
A8
F10
G9
SNU423
2B10
2D3
250
150
100
75
50
37
25
LATS2
β-Actin

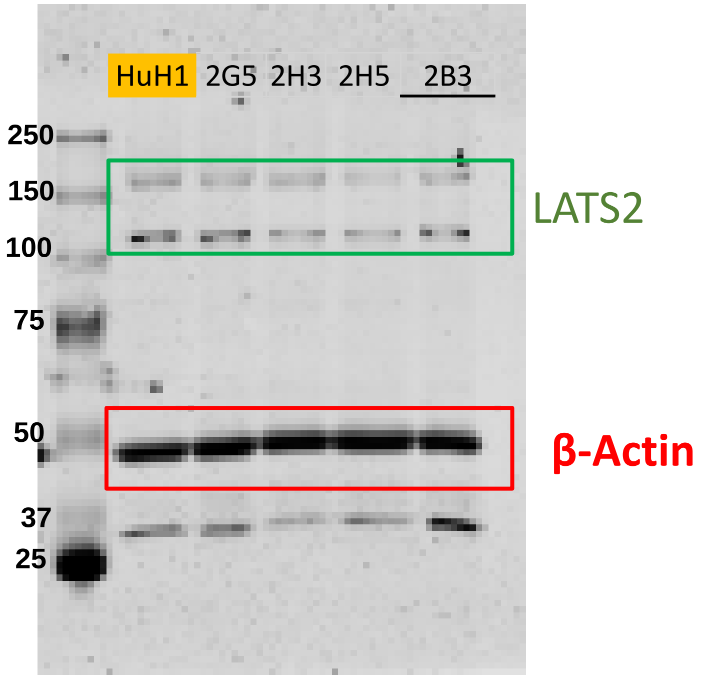

HuH1
2G5
2H3
2H5
2B3
250
150
100
75
50
37
25
LATS2
β-Actin

Supplementary Fig S2B

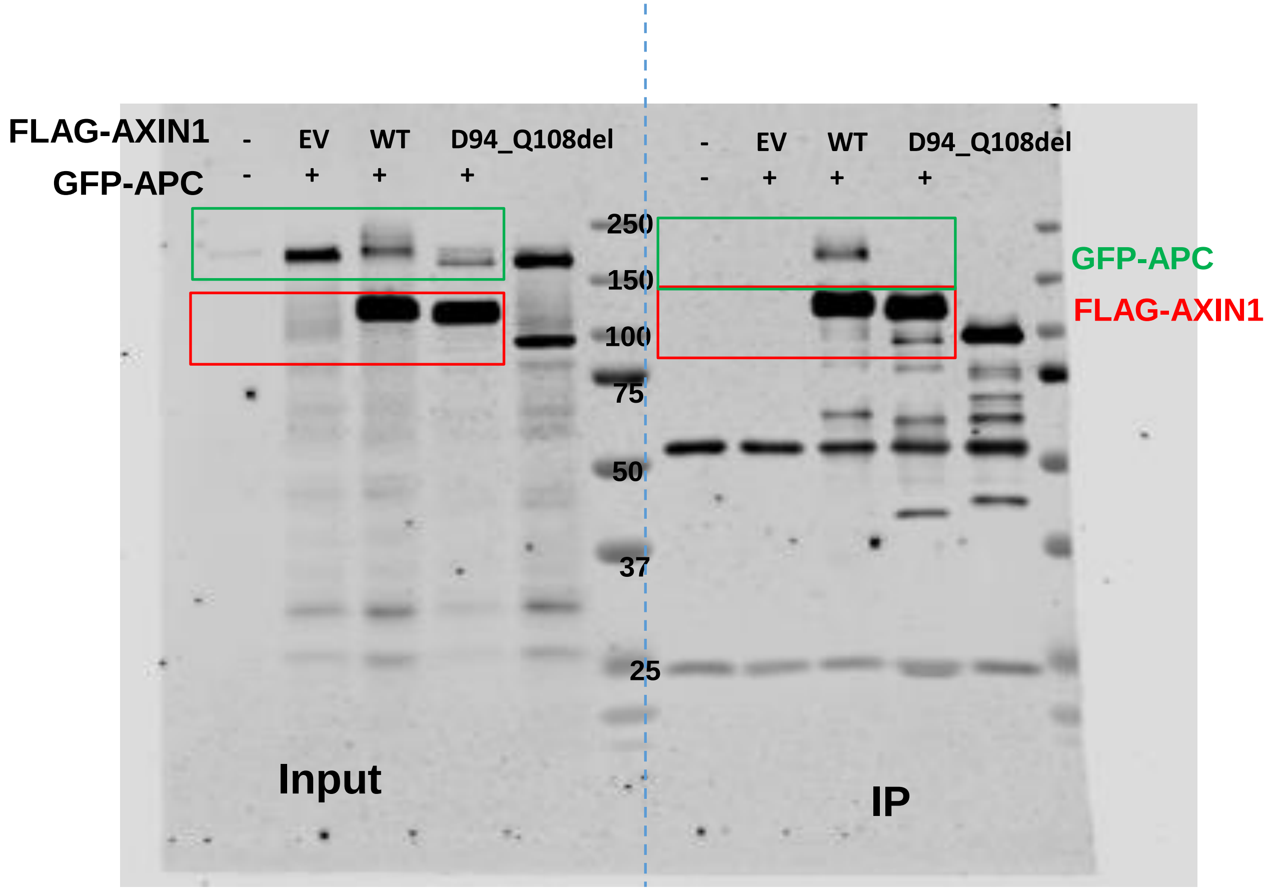

Supplement: S1 Raw images — (PDF) [file pone.0304607.s011.pdf]
