# Supplementary material for: Unraveling the impact of AXIN1 mutations on HCC development: Insights from CRISPR/Cas9 repaired AXIN1-mutant liver cancer cell lines
Source: PLoS One. 2024 Jun 7;19(6):e0304607. doi: 10.1371/journal.pone.0304607 (PMC11161089; doi:10.1371/journal.pone.0304607)
Supplement: S1 Table — (PDF) [file pone.0304607.s016.pdf]

Supplementary Table S1

Primer sequences for amplifying genomic AXIN1 Sequences and cloning into TA vector

Target	Primer name	oligos sequences
AXIN1 exon2	AXIN1-surEX2F	TGCAGCATTCTGACAGAGCAT
	AXIN1-surEX2R	ATGCCCTGAAACGTCCACT
AXIN1 exon3	AXIN1-surEX3F	GCAGAGACGTCTGAGCTGAAT
	AXIN1-surEX3R	GGCTGGGGTGTACATAGGTC
AXIN1 exon4	AXIN1-surEX4F	ACCATCACGTGTGAACCTTTG
	AXIN1-surEX4R	TTCTATGCAACAGCGAAAGCC
Primers to sequence AXIN1 products cloned into TA-vector	SP6	ATTTAGGTGACACTATAG
	T7	GTAATACGACTCACTATA
	M13F	ACTGGCCGTCGTTTTAC
	M13R	CAGGAAACAGCTATGAC
