# Supplementary material for: Unraveling the impact of AXIN1 mutations on HCC development: Insights from CRISPR/Cas9 repaired AXIN1-mutant liver cancer cell lines
Source: PLoS One. 2024 Jun 7;19(6):e0304607. doi: 10.1371/journal.pone.0304607 (PMC11161089; doi:10.1371/journal.pone.0304607)
Supplement: S2 Table — (PDF) [file pone.0304607.s017.pdf]

Supplementary Table S2 AXIN1 sgRNAs info

	gRNA primer	oligos sequences
Selected AXIN1 sgRNAs	JHH6-crF	caccgCCAAGGGGAAACCCTAATCT
	JHH6-crR	aaacAGATTAGGGTTTCCCCTTGGc
	JHH7-crF	caccgAGTCGGCACAGCCCTCCAGC
	JHH7-crR	aaacGCTGGAGGGCTGTGCCGACTc
	px458-hep3B mut-wt F	caccGAAGAATGTACTTTCAGTAGA
	px458-hep3B mut-wt R	aaacTCTACTGAAAGTACATTCTTC
	HuH1-crF2	caccgATAGCCGGCATTGACATAAT
	HuH1-crR2	aaacATTATGTCAATGCCGGCTATc
	SNU423-crF	caccgTCCCTGCGGTGCTGCTTACG
	SNU423-crR	aaacCGTAAGCAGCACCGCAGGGAc
Primer to confirm correct cloning of sgRNA oligos	px330-F	GATACAAGGCTGTTAGAGAG
