# Supplementary material for: Unraveling the impact of AXIN1 mutations on HCC development: Insights from CRISPR/Cas9 repaired AXIN1-mutant liver cancer cell lines
Source: PLoS One. 2024 Jun 7;19(6):e0304607. doi: 10.1371/journal.pone.0304607 (PMC11161089; doi:10.1371/journal.pone.0304607)
Supplement: S3 Table — (PDF) [file pone.0304607.s018.pdf]

Supplementary Table S3
Primers to introduce silent and PAM-site mutations in repair HDR plasmids

Target	primer name	oligos sequences
JHH6-AXIN1-exon2	JHH6-AXIN1-HDR-F	ATATtCAAGAgcAGGGTTTCCCCTTGGACCTC
	JHH6-AXIN1-HDR-R	TCATTTTGGGACTCTGCG
JHH7-AXIN1-exon2	Hep3b-ax1-repairF	TGGCGAGAGCcATCTACCGAA
	Hep3b-ax1-repairR	GCTTCAGCCTCTTCTCCT
Hep3B-AXIN1-exon2	AXIN1-kdL-Hep3B-F	taccGAAAGTACATTCTTGATAACAATGGCATC
	AXIN1-kdL-Hep3B-R	gatcGCTCTCGCCAGCTTCAGC
HuH1-AXIN1-exon3	HuH1-AXIN1-Q5-F	agtcaacCCgTAcTAcGTCAATGCCGgctatgc
	HuH1-AXIN1-Q5-R	ggctcccGCCAGGATCCATACCTGC
SNU423-AXIN1-exon4	SNU423-AXIN1-Q5-F	ggatccGTAAGCAGCACCGCAGGG
	SNU423-AXIN1-Q5-R	tgtatggGGGGATCCCATCCCTGTC
