# Supplementary material for: Unraveling the impact of AXIN1 mutations on HCC development: Insights from CRISPR/Cas9 repaired AXIN1-mutant liver cancer cell lines
Source: PLoS One. 2024 Jun 7;19(6):e0304607. doi: 10.1371/journal.pone.0304607 (PMC11161089; doi:10.1371/journal.pone.0304607)
Supplement: S4 Table — (PDF) [file pone.0304607.s019.pdf]

Supplementary Table S4
Primers to identify correctly repaired AXIN1 in cell clones

Target	Primer	oligos sequences
JHH6-AXIN1-exon2	JHH5/6-AXIN1-scF1	GAGAGGCTTTTCAGAGTCAAGTGC
	JHH6-AXIN1-scR	TCCAAGGGGAAACCCTgct
	JHH5/6-AXIN1-scR1	CCCAGGTCCAGATCCGAGC
JHH7-AXIN1-exon2	JHH7-AXIN1-scF	GAGACTTCGACGGCCACT
	Hep3B-AXIN1-scF1	GCTGGATGACCAAGATGGGAT
	Hep3B-AXIN1-scR2	CCAATTCTCCAATTCTTATCGATGC
HuH1-AXIN1-exon3	HuH1-AXIN1-scF	ACATACCGGCCCTGACAT
	HuH1-AXIN1-scR1	gTAcGGgttgactggctccc
	HuH1-AXIN1-scR2	TGAGGGACTGGGTATCCG
SNU423-AXIN1-exon4	SNU423-AXIN1-scF	GTAAATACCAGCTAGGAAGACGC
	SNU423-AXIN1-scR1	GCTGCTTACggatcctgtatgg
	SNU423-AXIN1-scR2	CTTTCCCGCGGACCAGTT
Hep3B-AXIN1-exon2	Hep3B-AXIN1-scF1	GCTGGATGACCAAGATGGGAT
	Hep3B-ori-F	CTGGCGAGAGCcATCTACt
	Hep3B-AXIN1-scR2	CCAATTCTCCAATTCTTATCGATGC
