# Supplementary material for: Unraveling the impact of AXIN1 mutations on HCC development: Insights from CRISPR/Cas9 repaired AXIN1-mutant liver cancer cell lines
Source: PLoS One. 2024 Jun 7;19(6):e0304607. doi: 10.1371/journal.pone.0304607 (PMC11161089; doi:10.1371/journal.pone.0304607)
Supplement: S5 Table — (PDF) [file pone.0304607.s020.pdf]

Supplementary Table S5
Primer sequences for amplifying AXIN1 cDNA

Primer name	oligos sequences
AXIN1-cdna3F	AACGACAGCGAGCAGCAGAG
AXIN1-cdna6R	AGCTTGTGACACGGCCCTGG
AXIN1-5UTR-F	GCGCTCATTGTTCCTTGACG
AXIN1-ex5R	CTTCGGCACCCGGTACG
