# Supplementary material for: Unraveling the impact of AXIN1 mutations on HCC development: Insights from CRISPR/Cas9 repaired AXIN1-mutant liver cancer cell lines
Source: PLoS One. 2024 Jun 7;19(6):e0304607. doi: 10.1371/journal.pone.0304607 (PMC11161089; doi:10.1371/journal.pone.0304607)
Supplement: S6 Table — (PDF) [file pone.0304607.s021.pdf]

Supplementary Table S6
Primer sequences used for Qpcr

Primer name	oligos sequences
GAPDH-F	GTCTCCTCTGACTTCAACAGCG
GAPDH-R	ACCACCCTGTTGCTGTAGCCAA
qpcr-h*HMMR*-F	GGCTGGGAAAAATGCAGAGGATG
qpcr-h*HMMR*-R	CCTTTAGTGCTGACTTGGTCTGC
qpcr-h*SPP1*-F	CGAGGTGATAGTGTGGTTTATGG
qpcr-h*SPP1*-R	GCACCATTCAACTCCTCGCTTTC
qpcr-h*HES1*-F	GGAAATGACAGTGAAGCACCTCC
qpcr-h*HES1*-R	GAAGCGGGTCACCTCGTTCATG
qpcr-h*TSPAN8*-F2	CATAGGCTTGCTTCTGATCCTGC
qpcr-h*TSPAN8*-R2	CTTTCCCCTGTGGCGCTCAAAA
AXIN2-qpcr-EX3F	TATCCAGTGATGCGCTGACG
AXIN2-qpcr-EX4R	TTACTGCCCACACGATAAGG
