# Supplementary material for: Unraveling the impact of AXIN1 mutations on HCC development: Insights from CRISPR/Cas9 repaired AXIN1-mutant liver cancer cell lines
Source: PLoS One. 2024 Jun 7;19(6):e0304607. doi: 10.1371/journal.pone.0304607 (PMC11161089; doi:10.1371/journal.pone.0304607)
Supplement: Attachment — Submitted filename: Response to reviewers PONE-D-23-31480.docx [file pone.0304607.s023.docx]

**Point-by-point response to reviewer’s comments**

Ref: PONE-D-23-31480

We thank both reviewers for their helpful comments.

**Reviewer #1**: In the manuscript the authors argue that they have successfully repaired AXIN1 utilising CRISPR/Cas9 mediated HDR in 5 HCC cell lines (JHH6, JHH7, Hep3B, HuH1, and SNU423). They show AXIN1 repair reduces b-catenin signalling, cell viability and colony formation. Their data suggest that Wnt3a stimulation of the repaired clones restored b-catenin activity and, in some lines colony numbers, but not overall cell growth. RNAseq data revealed cell-line specific responses to AXIN1 repair overlap were restricted to a small number of classic Wnt targets including AXIN2. Changes to AXIN1 did not appear to correlate with alterations to the Yap/Taz and Notch pathways despite prior data suggesting their involvement.

Although the data looks interesting, the results were somewhat frustrating to follow in their current state due to the lack of clarity in the methods describing how Wnt pathway activity was / was not maintained during the development and subsequent analysis of the clones being studied.

Major issue
The level of Wnt pathway activity was the key focus for the manuscript based on the hypothesis that restoration of Axin activity would downregulate TCF-dependent transcription. If the Wnt pathway were to behave as a digital ON/OFF switch, one might expect that the restoration of AXIN1 function would induce differentiation and perhaps prevent the emergence of clones. This possibility was mitigated by culturing the clones during selection in a combination of Wnt and Rspo conditioned medium. However, it was unclear whether 10% Wnt3a and Rspo CM was used to maintain AXIN1 repaired clones or whether the repaired clones were not viable without the CM.

>>> We apologize for not explaining our methods clearly enough. After first establishing in a simple trial experiment that the addition of Wnt3a and R-spondin did not clearly affect the growth of all five HCC cell lines, we only added it to the gene-editing experiment for safety. We hypothesized that if β-catenin signaling would be important for these lines and AXIN1 mutation was important to keep this at sufficiently high levels, we would fail to obtain repaired clones if β-catenin signaling was not maintained. Therefore, we added Wnt and R-spondin during establishment of the clones, but it was removed once we found correctly repaired clones and had sufficient back-up for all of them. We had expected, maybe hoped, that the clones would start to grow much worse without these additions, but this was not the case. After that we did all experiments in basal medium, except when specifically stated that Wnt3A was added for a specific analysis.

- To clarify this we have added the following sentence to the methods: “Once successfully repaired clones were identified, Wnt3A and R-spondin were removed from the medium and clones were further maintained in basal medium.”

The media conditions for the subsequent experiments were not made clear. Furthermore, Methods (Line 204) describes the use of RPSO CM alone and in combination with WNT3a CM however the results are only shown for Wnt3a CM– does addition of RSPO CM restore growth? A much more detailed discussion, exploration and explanation of the possible concerns / interpretation is required in addition to provision of the missing technical information.

>>> Mentioning R-Spondin conditioned medium in this part of the methods section resulted from a copy-paste from another manuscript. We accidently failed to correct these lines, which has been done now.

Suggestions to improve strength of conclusions
Although the observations (assuming the point above is adequately addressed) are interesting, they have to be interpreted with great caution due to the possible concerns covered here:

If the Wnt pathway were behaving as a variable-level rheostat (Cf. ON/OFF), there may be strong selective pressure for genetic or epigenetic modulation of Wnt pathway activity to maintain a ‘just right’ level for each individual cell type. Given that this interpretation is also consistent with the data, it would have been good to have seen the use of cell clones derived from a similar transfection / selection process to ensure that the selection methods used did not lead to variation in Wnt pathway activity in processes that were unlinked to the restoration of AXIN1 activity. Related to this point, studies could also have been done to show that reducing AXIN1 levels in the clones (e.g. using shRNA or siRNA) reversed the effects of the rescue since this would exclude the ‘clonal variation’ explanation.
>>> We understand the concern of the reviewer, but we hope that he/she also appreciates that we have obtained 14 independent AXIN1-repaired clones from 5 different cell lines that in all cases show reduced growth characteristics associated with reduced β-catenin signaling. It is very unlikely that this is the result of clonal selection for “just-right” β-catenin signaling levels.

In support of this is an observation that we made during establishment of these clones. Following transfection of Crispr/Cas gene editing plasmids, clones started to emerge. When they reached a sufficiently big size, they were picked. The first batches of clones rarely yielded AXIN1-repaired clones, which were mainly obtained several days later. This was actually the first indication that AXIN1-repaired clones expanded slower.

Admittedly, on hindsight it would have been better to include some non-repaired clones in our analysis. We have also played with the idea to knockdown repaired AXIN1 again using siRNA, but we reasoned that using siRNA, this will only be a temporary and partial knockdown, which may not be sufficient to see clear changes in 7-days MTT or colony formation assays. Combining the consistent observations for the 14 established clones with pragmatic and budgetary reasons, we hope the reviewer will agree with us that such an undertaking is very unlikely to affect the take-home messages from our paper on reduced growth characteristics and β-catenin signaling.

It would also have been useful to have seen some studies carried out using purified Wnts / Rspo proteins and efforts to monitor levels in the culture media since some of the variable responses described between cell lines might be related to the rates at which active Wnt/Rspo-ligands are maintained / depleted in culture.
>>> We agree with the reviewer that it is difficult to completely control how Wnt and Rspondin exposure affects the cells during the time course of the experiment. We have aimed at adding Wnt levels that restore β-catenin signaling to levels similar to the ones observed in the parental cells, based on β-catenin reporter activity. However, these ligands are added to the cells at one time point and then refreshed a few days later. One expects that shortly following addition, β-catenin signaling is increased in most cells. How long that increased signaling level is maintained, is difficult to state with certainty, but for example based on the β-catenin reporter activity, we expect that it will also be higher than baseline even a few days after addition (β-catenin luciferase reporter activity was measured 48 hours after addition of ligands. Luciferase half-life in cells is only about 3 hours. As we still see clearly increased levels 48 hours later, the luciferase that we measure at that time point is very likely also coming from enhanced luciferase production close to the time that the cells are analyzed. In other words, this suggests that also at that time there is still increased β-catenin signaling resulting from the ligands added).

That shortcoming is also expected from purified Wnts and Rspondin. Also in that case it will be difficult to completely control the level of signaling during a few days. Likewise, for the use of GSK3β inhibitors that we also have considered. Besides that it is more difficult to fine-tune the level of β-catenin activation accomplished using these inhibitors, it is also expected to affect other cellular processes regulated by GSK3β.

About the link between AXIN1 mutation and β-catenin signaling; reviewer 2 pointed out a paper in which 4 out of 5 cell lines tested by us (all except SNU423), were treated with siRNA to knockdown β-catenin. Except for Hep3B that was reduced in its growth, the other three were not affected by β-catenin knockdown. This supports our observations that β-catenin signaling levels are not the only explanation for the tumorigenic potential of AXIN1 mutation. We have added this paper to the discussion.

We have the feeling that the reviewer shares the same frustration as us when conducting this research project. We established 14 AXIN1-repaired clones, showed that they are all decreased in their growth capacity, which is accompanied by a clearly reduced level of β-catenin signaling. Based on this we were hoping for an easy answer, that is to demonstrate that the reduced growth capacity can be explained by the alteration in β-catenin signaling, but this was not the case or at most partially. Following that first disappointment, we have tried to find an alternative common explanation linked to AXIN1 mutation, but again failed to find a straightforward explanation. After that we decided to share all this information, hence its submission to PLOS One.

Minor points:
How was the AXIN1 antibody determined to be against C-terminus of the protein
>>> This is information that we obtained from the Cell Signaling Technology company. The first time that we asked we wished to know why it fails to identify the R712* truncating protein present in SNU449. We were then told that the proprietary epitope is after this amino acid. The second time we were told that the epitope is between Q630 and E760. Combining both it would indicate that the epitope is between 712-760, which is quite C-terminal in the AXIN1 protein. This information is added to the methods section now.

Line 277-278 How homozygosity or heterozygosity of repair was not explained. And no sequencing data presented to show the repair of AXIN1.

>>> Homo- or heterozygous repair was determined by first performing a PCR on genomic DNA of repaired clones, using at least one primer outside of the targeting construct (this to prevent that we are looking at clones with random integration of the repair plasmid). Sequence analysis of these PCR products then clearly shows either a uniform repair of the original mutation (so clean peaks with the expected base modifications) or heterozygous peaks. We confirmed this also on cDNA of the same clones as we occasionally see that the genomic alterations do not entirely match the cDNA alterations. We have now added these sequence analyses as supplemental figure S3.

While making these figures we also noticed that 1 out of 4 repaired HuH-1 clones (clone 2B3) actually carries one chromosome that is not repaired. The other two chromosomes are correctly repaired. However, on cDNA level only the repaired transcript is observed, which can possibly be explained by nonsense-mediated decay of the mutant transcript. We have now also corrected this in the results. Importantly, this alteration does not affect the conclusion as this clone shows a clear expression of repaired AXIN1.

Figure 2 B, C, D cell line labels missing above most of the charts.

>>> We have now added this to all panels. The order is the same as in panel A.

Figure 4 A β-catenin signalling activity missing statistics.

>>> Although this is not a trivial issue here, because which statistical comparison do you make, we have decided to add statistics comparing Wnt3a treated values for each repaired clone with their corresponding non-treated counterpart.

Figure 4 B is missing error bars

>>> These have been added now.

Figure 6 C. Quantification of protein levels required

>>> This we have done now and added it as a supplemental figure S10.

Additionally, keep order of cell lines same as in previous figures (SNU423 and HuH1 blots switched)

>>> This has been adjusted.

Methods describe assays not shown in the results:

Statistical analysis – methods state two tailed students t test used to compare between two groups while non parametric test – Mann-Whitney U test used for groups with non-normally distributed variables. Figure legends indicate T Test not used once. Not clear what data points are being shown in charts – is it n in each experiment or the mean from each experiment.

>>> we have checked which tests were used and adapted the methods section accordingly. In all cases we show mean ± SD. This is mentioned in the figure legends.

Line 378-379 KEGG analysis – data not shown?

>>> We have now added this as supplemental figure S8.

Line 164-167 – Methods indicate use of fluorescent western blotting – no fluorescent blots shown in results

>>> Several western blots are done using fluorescent western blotting (for example the ones in Figure 6), but we present them as gray-scale images. Maybe this created the confusion that we did not use this technique. All original western blots are now included as supplemental figure S11.

Sup Table G qPCR – primers for Axin1 exons 7-8. No AXIN1 expression data shown. Also SERPINE1 primers but no data

>>> We have removed these from the table text.

**Reviewer #2**: In the manuscript “Unraveling the Impact of AXIN1 Mutations on HCC Development: Insights from CRISPR/Cas9 repaired AXIN1-mutant liver cancer cell lines”, Zhang et al examined phenotypes of 5 human HCC cell lines with restored wild-type AXIN1. The study examined 2-4 clones for each cell line and found reduced beta-catenin activity, cell viability, and colony formation. Restored beta-catenin activity with Wnt stimulation did not overcome growth defects, suggesting that activities other than beta-catenin are involved in this phenotype. Canonical Wnt target genes were not consistently altered in each of the rescued clones, again consistent with effects of AXIN1 mutations beyond the regulation of beta-catenin signaling. Further, RNAseq and western blot analysis did not support a consistent pathway regulated by AXIN1 in each line, including YAP/TAZ or Notch.

Though the manuscript is clearly written and logical, some experiments were not conducted rigorously, with appropriate replication, and sample sizes. For Fig. 2D, Fig. 3B, Fig. 4 A and B, Fig. 5, and Fig. 7B, the experiments were only performed two independent times. Performing three times is required to increase rigor.

>>> The reviewer is correct that repeating an experiment three times will increase rigor. However, both for pragmatic as well as budget reasons, we decided to repeat some experiments only two times. In all cases where we chose that option, both independent experiments yielded the same conclusion, so we are quite confident in these results. For example, for figure 2D in which we knockdown AXIN2 using Smartpool reagents, in basically all clones with AXIN1 repair the β-catenin luciferase reporter was only modestly increased, while that of the parental AXIN1-mutant cell line was strongly increased. Each reporter experiment was performed with 6 replicates per clone and condition. In this case the differences between parental and repaired clones are so obvious that doing the experiment just once would have yielded a good conclusion. Nevertheless, we decided to repeat it once with comparable results. Similar reasoning applies to the other experiments pointed out by the reviewer.

The authors have made some of the data supporting the findings in their manuscript available. However, other data should be expanded to help readers more fully understand and interpret the unexpected results with the cell phenotypes. Notably, the experimental details for how the AXIN1 mutations in the HCC lines were identified and confirmed are lacking. Some of the mutations differ from those described in publicly available databases such as “the Liver Cancer Cell Line Database”. The precise mutations as well as their hetero- or homozygosity become important when considering potential explanations for the unexpected results.

>>> After having had some bad experiences with miscalled genomic alterations in cell lines based on database information, we always first extensively confirm the exact nature of a mutation before initiating a gene-editing experiment. We have now described our procedure in the methods section (PCR followed by Sanger sequencing), and also included the chromatograms as in Supplemental Figure S1B.

This shows that all identified mutations are homozygous, information that cannot easily be retrieved from the liver cancer cell line database. The homozygous nature also matches with the lack of an identifiable protein using a more C-terminal AXIN1 antibody (except for JHH7 that carries an N-terminal **internal** deletion).

Indeed, we have two “deviations” from the database:

- For JHH6 we forgot to mention that the Q6* stop codon mutation is preceded by an E5D missense variant. We have now added that to Supplemental Figure 1 and Figure 1A.

- For Huh1 the frameshift ends after 112 amino acids, not the 110 that we originally mentioned. This has also been corrected. The Liver Cancer Cell Line Database mentions a R298L and E299Pfs*111 mutation for this line. In our opinion it is more correct to refer to it as R298Lfs*112.

Another dataset that would increase the rigor of the study is to show the complete length of the western blot of the parental cell line lysates probed with both a C-terminal and an N-terminal AXIN1 antibody (Fig. 2C). This would support the statement on line 255 that the mutations lead to short, truncated proteins. Likewise, it would be good to show more of the gel and molecular weight markers to confirm the size of the restored Axin1 product in Fig. 2A. If these blots were probed with an N-terminal AXIN1 antibody it could be demonstrated that truncated AXIN1 was eliminated in all but SNU423 cells (as stated in the text).

>>> For all western blots we have now added the original full blots including the size marker as Supplemental Figure S11. In the past we had an N-terminal AXIN1 antibody available from Cell Signaling Technology (#3323). However, that antibody has been discontinued by this company. This antibody did recognize AXIN1, but also showed some weak additional bands, which is maybe the reason it is not available anymore. We don’t have this antibody anymore in the lab, and attempts to find a suitable replacement failed so far. As this means that we cannot show a blot probed with an N-terminal antibody, we have adapted line 255 as follows:

“all carry mutations **predicted** to lead to short truncated proteins lacking most functional AXIN1 domains”

The level of Axin1 restored appears to be quite variable- eg. HuH1 and SNU423 are quite faint. Are the restored Axin1 levels comparable to endogenous levels in HEK203T, or other HCC lines with wild-type Axin1?

>>> Although all five cell lines are considered liver cancer cell lines, they have many characteristics that are expected to be different. In this case, most of the variation is in line with *AXIN1* RNA expression levels that we retrieved from the protein atlas database. SNU423 and HuH1 are the two cell lines with lowest level of *AXIN1* RNA expression (JHH6 24.3 nTPM; JHH7 21.9; Hep3B 18.4; HuH1 11.8 and SNU423 6 (nTPM= normalized transcript per million)). Importantly, the expression levels of repaired AXIN1 protein are sufficient to inhibit β-catenin signaling in all cases, showing that they are expressed at functionally relevant levels.

Fig. 2D has a label “JHH7” in the middle of the panel.

>>> Our original image had cell line names in all panels, but because this led to a “noisy” image we removed all of the names, except for one that we forgot. Reviewer 1 wishes to see these name labels, so we have now placed them back to all panels.

Why is the GFP-APC band shifted in supplementary fig 2B depending on which FLAG-AXIN1 it was immunoprecipitated with?

>>> This is very well-spotted by the reviewer. The apparent shift in size is resulting from GSK3β and CK1-mediated phosphorylation of APC. It is already known for some time that when AXIN1 and APC can form a functional complex, GSK3β and CK1 bound to AXIN1 will also phosphorylate several residues in the β-catenin regulatory domains of APC. This will strengthen the binding to β-catenin, but the additional phosphate groups will also lead to an upward shift of APC in the gel. As the D94_Q108del AXIN1 variant cannot bind to APC, this phosphorylation does not occur and GFP-APC will remain low, while for wild-type AXIN1 this phosphorylation occurs leading to this upward shift. In our recent Cancer Research paper on AXIN1 (PMID 38359148) this upward shift is also seen consistently for variants that efficiently bind to APC, but not for the ones that show no or reduced binding (Figure 5B)).

Fig. 1D Are WRE and MRE both controlled for transfection (CMV-renilla) when presented as WRE/MRE?

>>> Yes, both WRE and MRE values are first normalized for CMV-Renilla.

The level of AXIN2 mRNA is not significantly reduced in the AXIN1-repaired clone JHH6-E12 (in contrast to line 282 statement) which also showed no decrease in NOTUM mRNA level (sup Fig. 3).

>>> The reviewer is correct. For AXIN2 we have now added the following text: “except for JHH6 clone E12”. For Notum this statement was already present in the text.

How is the large (1000x) range of results explained in Fig. 2D? In other words, why is there a ~5-fold difference in beta-catenin reporter activity with AXIN2 knockdown in parental Hep3B cells and a ~5000-fold difference in parental HuH1 cells?

>>> We cannot fully explain this, but we feel that it is again inherent to working with different cell lines. Each cell line will be different, for example, in how well it can be transfected with the siRNAs or how much *AXIN2* mRNA must be knocked down. This and many other features will affect the final activation of the WRE reporter. It is also important to realize that this is a sensitive reporter that more readily picks up differences in β-catenin signaling than most other methods. Occasionally, this leads to a very high induction of reporter activity.

The statement in line 292 seems to miss the mark—the AXIN1 mutant HCC cells appear more dependent on AXIN2 for beta-catenin regulation?

>>> We believe that the reviewer and we agree on the final conclusion, but have used slightly different words for it. We have carefully looked at our text to make it more clear and now use this text: “This confirms that AXIN1 mutant HCC cells are strongly dependent on AXIN2 to counterbalance signals that induce β-catenin signaling, and are more vulnerable to aberrantly increase this signaling pathway”.

The reason to bring up the vulnerability issue is that we wish to indicate that AXIN1-mutant cells will have more difficulty to keep β-catenin signaling in check, despite that AXIN2 can partially compensate. In a group of AXIN1-mutant tumor cells, we speculate that occasionally some cells will have low AXIN2 activity. These cells will then have more β-catenin signaling than wild-type cells, which maybe gives them some advantage in an inflamed and cirrhotic liver (most HCCs emerge in patients with chronic liver damage). As this is speculative we do not describe it in our manuscript.

For Fig. 2D, the values are shown “relative to the WRE/CMV-Renilla ratios obtained for the siControl-WT which is set to 1” However, there doesn’t appear to be anything shown as “1” in this figure or in Sup Fig 4. It is unclear how these values were obtained and what they mean.

>>> We understand that this may be confusing. For that reason we have extended our text on this in the legend to better indicate what we have done as follows: “WRE/CMV-Renilla ratios were obtained for siAXIN2 and siControl for each clone. Next, for each clone this WRE/CMV ratio was set to 1 for the siControl value, to which the siAXIN2 value was normalized. Finally, the figure shows the normalized siAXIN2/siControl ratio (mean ± SD, n=6, two independent experiments).“

- To use JHH6 as an example with arbitrary numbers. In the parental clone the β-catenin reporter activity is for example 1 when treated with siControl. This reporter activity increases more than 40-fold when treated with siAXIN2. For both repaired clones, the reporter activity is about half of the parental cells when treated with siControl. This increases to about 1.5 when treated with siAXIN2, so a 3-fold increase. The 40 and 3-fold increases are then shown in Figure 2D, while supplemental figure S4 shows the real WRE/CMV-Renilla values obtained for each clone and each siRNA treatment (see also explanation below).

For sup Fig. 4, the X-axis is shown on a logarithmic scale, but the values themselves are not log10. The axis labeling is confusing on these graphs and those in Fig. 4A.

>>> What we depicted here are WRE/CMV-Renilla ratios as log10 values. Maybe the confusion arose because in this case the CMV-Renilla values are often higher than those of WRE reporter. Let’s again use the JHH6 parental line treated with siControl as an example. If the WRE yielded 5.000 luciferase light units and the CMV-Renilla 100.000, the WRE/CMV ratio that we obtained would be 0.05. This value is then depicted on the Y-axis. In this case we chose to use a log10 scale to depict these values as otherwise the differences in bar height would become too big, and low values would not be visible anymore as bars. Personally, we also prefer absolute numbers as differences are more intuitive, but in some cases like when differences in values are too big, we unfortunately need the logarithmic scales. In this new version, we maintained the logarithmic scale for Sup Fig S4, but replaced the ones for Fig 4A for non-logarithmic values.

In Figure 3A and 4B, the growth rate is not being measured. With more time points between d3 and d7, the growth rate could be estimated and one could determine if 1) there is a lag until d3 and then all lines start to grow, but the parental grows more quickly or 2) there is a longer lag with the rescue clones and they are less able to grow in sparse conditions (consistent with the colony formation assay).

>>> The reviewer is correct that adding more time points would allow better to determine a growth rate value, but the take-home message that AXIN1-repaired clones expand slower, will not change. We have little doubt that repairing AXIN1 in these cell lines resulted in reduced growth characteristics. First, during establishment of the clones they were picked on different time points after transfection depending on their size (bigger clones first). Basically without exception, the repaired clones were picked at later time points, meaning that it took them a longer time to get to a clone size that we could pick from the plate. Second, during routine culture of these clones, it always took the repaired ones more time to fill the flask. In fact, we started passaging them at different ratios (like 1:5 for the repaired clones, 1:10 for the parental ones) to have full flasks again at more or less the same day. These day-to-day observations cannot be used for publication obviously, hence the MTT and colony formation assay, but we hope that the reviewer agrees that the take-home message will not be critically altered by adding a few more time points.

Line 319- “suggest that mechanistically this is accomplished by increasing…”—no mechanism is shown, but there is a correlation with beta-catenin signaling.

>>> We mean the same as the reviewer, but indeed our original choice of words could be misinterpreted. We have changed it to:

“Thus, these findings demonstrate that AXIN1 mutation confers a clear growth advantage onto liver cancer cells, which correlates with increased β-catenin signaling.”

Line 347: “we cannot formally demonstrate that reduced beta-catenin signaling is also responsible for the observed change” seems misleading. Beta-catenin activity with added Wnt does not correlate with growth for every cell line. In some cases (eg. JHH6) adding Wnt results in fewer colony numbers.

>>> Following these remarks by the reviewer, we have critically looked at our text accompanying figures 4 and 5. We feel that our new text provides a better description of the results in which sometimes a partial response is observed in the MTT or colony formation assay. In our original text, we described it more in black-white terms, while the current version provides a more balanced view.

For Fig. 6C, are these one blot for each cell-line set that was probed with 10 different antibodies? Were these blots stripped and re-probed? More experimental details in the methods section would be useful.

>>> As for all the other western blots, we have now included the original blots as supplemental images. No re-probing of blots was done.

The experimental results are intriguing and unexpected. The authors should consider if the results in Fig. 5 are consistent with Ding et al, Oncotarget, 2017 “Oncogenic dependency on β-catenin in liver cancer cell lines correlates with pathway activation” who found that silencing beta-catenin led to growth defects in Hep3B cells but not in JHH6, JHH7 or HuH1 (supplementary Fig. 6, 10, 12, and 17). Can other properties of the 5 parental cells be considered to obtain more information from the data? For instance, 3 are from Japanese patients, 3 of the cell lines are associated with hepatitis virus infection, four have P53 mutations and 3 have TERT mutations.
>>> We thank the reviewer for pointing out this reference, which skipped our attention. We have now added it to the discussion to indicate that not all AXIN1-mutant HCC cell lines are equally sensitive to alterations in β-catenin levels.

As for the suggestion to check other properties such as P53 or TERT mutation. This may theoretically indeed influence the outcome of the growth characteristics that we observe, but as we have only looked at 5 cell lines, it will be impossible to find any meaningful correlations.

Overall, this is an interesting study with intriguing results. The results are worth presenting in the most robust form possible, particularly since they contrast with other published results.
